# Dietary Inclusion of Seabuckthorn (*Hippophae rhamnoides*) Mitigates Foodborne Enteritis in Zebrafish Through the Gut-Liver Immune Axis

**DOI:** 10.3389/fphys.2022.831226

**Published:** 2022-04-06

**Authors:** Ming Li, Xuyang Zhao, Jiayuan Xie, Xinyu Tong, Junwei Shan, Mijuan Shi, Guangxin Wang, Weidong Ye, Yuhang Liu, Bruno Hamish Unger, Yingyin Cheng, Wanting Zhang, Nan Wu, Xiao-Qin Xia

**Affiliations:** ^1^ Institute of Hydrobiology, Chinese Academy of Sciences, Wuhan, China; ^2^ College of Fisheries and Life Science, Dalian Ocean University, Dalian, China; ^3^ College of Advanced Agricultural Sciences, University of Chinese Academy of Sciences, Beijing, China; ^4^ Hubei Key Laboratory of Agricultural Bioinformatics, College of Informatics, Huazhong Agricultural University, Wuhan, China

**Keywords:** herb therapy, anti-inflammation, intestinal microbiota, fatty acid metabolism, immune crosstalk

## Abstract

To help prevent foodborne enteritis in aquaculture, several feed additives, such as herbal medicine, have been added to fish diets. Predictions of effective herb medicines for treating fish foodborne enteritis from key regulated DEGs (differentially expressed genes) in transcriptomic data can aid in the development of feed additives using the Traditional Chinese Medicine Integrated Database. Seabuckthorn has been assessed as a promising candidate for treating grass carp soybean-induced enteritis (SBMIE). In the present study, the SBMIE zebrafish model was used to assess seabuckthorn’s therapeutic or preventative effects. The results showed that intestinal and hepatic inflammation was reduced when seabuckthorn was added, either pathologically (improved intestinal villi morphology, less oil-drops) or growth-related (body fat deposition). Moreover, seabuckthorn may block the intestinal p53 signaling pathway, while activating the PPAR signaling pathway and fatty acid metabolism in the liver. 16S rRNA gene sequencing results also indicated a significant increase in OTU numbers and skewed overlapping with the fish meal group following the addition of seabuckthorn. Additionally, there were signs of altered gut microbiota taxa composition, particularly for reduced TM7, *Sphingomonas*, and *Shigella*, following the addition of seabuckthorn. Hindgut imaging of fluorescent immune cells in SBMIE larvae revealed the immune regulatory mechanisms at the cellular level. Seabuckthorn may significantly inhibit the inflammatory gathering of neutrophils, macrophages, and mature T cells, as well as cellular protrusions’ formation. On the other hand, in larvae, seabuckthorn inhibited the inflammatory aggregation of lck^+^ T cells but not immature lymphocytes, indicating that it affected intestinal adaptive immunity. Although seabuckthorn did not affect the distribution of intestinal CD4^+^ cells, the number of hepatic CD4^+^ cells were reduced in fish from the seabuckthorn supplementation group. Thus, the current data indicate that seabuckthorn may alleviate foodborne gut-liver symptoms by enhancing intestinal mucosal immunity and microbiota while simultaneously inhibiting hepatic adipose disposition, making it a potential additive for preventing fish foodborne gut-liver symptoms.

## Introduction

Due to the high cost and limited supply of fishmeal, the increased inclusion of plant proteins in aquafeeds has become a common practice (Li et al., 2019). Given the importance of plant proteins as fish meal substitutes in aquaculture, consequential side effects such as foodborne enteritis and hepatic symptoms should be avoided or treated. Though carnivorous fish species are much more sensitive to plant-sourced proteins like soybean meal (SBM) (Booman et al., 2018), herbivorous and omnivorous species like grass carp (*Ctenopharyngodon idella*) and zebrafish (*Danio rerio*) can also develop enteritis and liver pathologies (Wu et al., 2018; Wu et al., 2020). Recently, additives or probiotics have been used to prevent decreased digestion, enteritis, and poor growth performance caused by over-substitution with plant proteins.

With advancing transgenic techniques, fluorescent proteins can be used with zebrafish lines to label various immune cell populations, including T cells (Kasheta et al., 2017; Coronado et al., 2019; Li et al., 2020a), macrophages (Ellett et al., 2011) and neutrophils (Brugman et al., 2009; Oehlers et al., 2011; Okuda et al., 2015; Enya et al., 2018). At approximately 1 week of age (6 to 9 dpf), the early phases of intestinal innate immune cells, including neutrophils and macrophages, can be detected (Bravo-Tello et al., 2017; Ikeda-Ohtsubo et al., 2020). At an adaptive level, intestinal lymphocytes, including mainly T and B lymphocytes can also be observed at a very early age (5 dpf), and later the response related to T cell subpopulations could be observed in 3- to 4- week-old larva (before intestinal pigments cover) (Coronado et al., 2019). Thus, zebrafish may be an excellent model organism for screening and demonstrating the processes of innovative and effective additions.

Additionally, computational approaches to medicine selection have shed light on veterinary research and its applications. Herb medicines have been used to treat animal diseases and can occasionally be used in place of antibiotics to improve animal health. The bioinformatics database TCMID (Traditional Chinese Medicines Integrated Database) contains comprehensive information on the interactions of compounds, proteins and herbs (Qu et al., 2019a; Zhang et al., 2020a). The database can provide formula, herbal medicine, herbal ingredients, and herbal ingredients-target information. In aquaculture, herbs have also been used to enhance fish immunity and growth performance (Zhang et al., 2009; Abarike et al., 2019).

Dietary supplements containing *Astragalus membranaceus, Angelica sinensis*, and *Crataegus hupehensis* can increase the expression of immune genes such as beta-defensin, lysozyme, and heat shock protein 70 in both the intestine and the head-kidney, enhancing growth performance and disease resistance in tilapia (*Oreochromis niloticus*) (Abarike et al., 2019). Propolis and *Herba Epimedii* extracts may promote respiratory burst activity, phagocytosis of phagocytic cells in the blood, and lysozyme activity in plasma in Chinese suckerfish (*Myxocyprinus asiaticus*) (Zhang et al., 2009). As a result of its widespread usage in systematic pharmacology and the recent findings on effective herbs (Zhang et al., 2020b; Wang et al., 2021; Ye et al., 2021; Yu et al., 2021) for treating enteritis (Yu et al., 2021), TCMID may also serve as a platform for predicting successful medications or feed additives in aquaculture.

The protective effect of dietary ingredients affects both the gut and liver of fish via the biofluid network (Roques et al., 2020). This includes bile acid, intestinal mucus, and microbiota, as well as metabolites and immune cells found in the blood. Hepatic disease can result in dysfunction of the intestinal barrier and host-microbiome imbalances (Tranah et al., 2021). Thus, the fish gut-liver immune axis has been proposed (Wu et al., 2016) and has been demonstrated to be involved in the development of foodborne gut-liver symptoms (Wu et al., 2018) in a variety of commercial species. While zebrafish are a commonly used animal model in aquaculture research, it was discovered that hepatic health also affects the intestine mucosa. For instance, the hepatotoxicity of palmitic acid alters the intestinal microbiota (Ding et al., 2018).

Gut microbiota are essential for the gut-liver axis’ immune homeostasis (Yang et al., 2020). Diet may have a significant impact on the complex interplay between the gut microbiota, the intestinal barrier, the immune system, and the liver (Martín-Mateos and Albillos, 2021). In fish, the gut microbiota is involved in intestinal barrier function, such as intestinal immune reactions and intestinal integrity (Li et al., 2019). The fact that not only effective additives but also fermented soybean meal (FSBM) are utilized to complement the diet may help alleviate foodborne enteritis in the fish (Catalán et al., 2018; Li et al., 2019; Liu et al., 2019; Li et al., 2020b), indicating the bacteria’s functional role in intestinal homeostasis. FSBM was discovered to improve the health and growth physiology of salmon by increasing the growth of intestinal lactic acid bacteria, resulting in a prebiotic-like effect (Catalán et al., 2018). SBM fermented with *Enterococcus faecium* (Li et al., 2020b) or the addition of the commensal bacterial *Shewanella* sp. MR7 (Li et al., 2019) is effective against the typical SBMIE (soybean meal induced enteritis) in turbot. Supplementation of *Bacillus*’ metabolite sodium butyrate in a high-soybean meal fish diet (40% fish meal protein replaced by soy protein) may alter the composition of the intestinal microbiota whilst regulating intestinal gene expression, such as by downregulating TNF-alpha and NF-kappaB and upregulating genes involved in the intestinal epithelium’s tight junction (Liu et al., 2019).

Seabuckthorn (*Hippophae rhamnoides*), a traditional Mongolian and Tibetan medicine, has been shown to protect against liver disease by regulating the liver metabolome and abundance of gut microbiota (Ran et al., 2021). Its protective effect on the liver has been well established, not only in diet-induced but also in toxicant-induced hepatic symptoms (Kwon et al., 2017; Wang et al., 2018; Zhao et al., 2020). However, research on its intestinal effect is still in its infancy. As a result of the gut-liver axis, the gut microbiota may be influenced, when seabuckthorn supplementation in the diet increases the ratio of Firmicutes/Bacteroidetes (Guo et al., 2020). For treating intestinal damage or inflammation, seabuckthorn oil downregulated intestinal mRNA levels of inflammatory factors, including TNF-α, IL-1β, IL-6 and IL-8 in mice (Shi et al., 2017). Additionally, seabuckthorn berry juice may increase the antioxidant capacity of rats in a simulated gastrointestinal tract (Zhao et al., 2020). As a result, seabuckthorn was hypothesized to affect fish gut-liver symptoms. TCMID was used to screen drugs for SBMIE in this study, and it was predicted that seabuckthorn, as well as the target genes, may aid in the treatment of fish SBMIE.

## Materials and Methods

### Prediction of Effective Herb Against Fish SBMIE

The current study used the TCMID (http://www.megabionet.org/tcmid/) to predict the target drug to investigate in the treatment of fish enteritis. The STRING database was used to obtain information on protein-protein interactions. To identify key fish SBMIE genes, we used the Page Rank algorithm to access previously published grass carp SBMIE transcriptomic DEG data (Wu et al., 2018), which included gene ID, fold change, and expression level (Morrison et al., 2005). The importance of a gene in this algorithm was determined by the network’s structure and its initial importance. The structure of the network can be determined by the gene dependence network, and the initial significance of the gene can be determined by its differential expression.

Fold-change was used to calculate its differential expression: *fd* = log_2_ *a −* log_2_ *b*. Where “a” was the expressional value of a gene in the diseased intestine and “b” was the expressional value of the gene in the control. Therefore, when *fd* > 0, it showed that the gene was upregulated by *fd* times in disease tissues, and if *fd* < 0, it showed that the gene was downregulated by *fd* times. On this basis, the following formula was used to calculate the importance of each gene: $r_{j}^{n}=\left(1-d\right)ex_{j}+d\sum_{i=1}^{N}\frac{w_{ij}r_{i}^{n-1}}{\deg_{i}}$ (Morrison et al., 2005). Where “ $r_{j}^{n}$ ” was the calculated importance value of gene “j.” “ $ex_{j}$ ” was the initial value of gene “j.” “ $w_{ij}$ ” was the dependence in the gene dependence network, if gene “i” depended on gene “j,” then $w_{ij}$ = 1, otherwise it was 0.“ $r_{i}^{n-1}$ ” was the value of the “i” gene after the “n−1” iteration. “ $\deg_{i}$ ” was the degree of the vertex “i.” The parameter “d” (0 < = d < 1) was a constant, and “d” represented the proportion of gene dependence in the calculation process.

The formula indicated that the relevance of vertex “j” was dependent on two values: the initial importance of the gene (the differential expression value of gene I and the importance of all vertices pointing to vertex “j” (the second term on the right of the formula). If “d” was greater, it indicated that the genes’ importance was more dependent on gene dependence. When “d” was less than one, it indicated that the genes were more reliant on their initial importance. In this case, “d” prioritized 0.5. The DEGs (differentially expressed genes) of SBMIE can be sorted according to their relevance from highest to lowest using the above calculation.

The top 300 Generank-ranked genes in grass carp SBMIE DEGs (Wu et al., 2018) were used as the drug’s target genes, and the herbal components that can target these genes were determined using the TCMID database’s information on the relationships between effective compounds and genes (Huang et al., 2018). Herbal medicines containing the herbal ingredients discovered using the aforementioned methods were mined using the TCMID database’s information on herbal ingredients. The top herbal medicine was chosen for follow-up experimental verification based on the number of ingredients.

### Zebrafish, Diets and Feeding Trial

In the current study, wild-type zebrafish (AB strain), as well as several ZFIN (the Zebrafish Information Network, http://zfin.org/) included transgenic zebrafish lines, including *Tg(lyz:DsRED2)* (with neutrophils labeled, https://zfin.org/ZDB-TGCONSTRCT-071109-3), (Campbell et al., 2021), *Tg(mpeg1:EGFP)* (with macrophages labeled, http://zfin.org/ZDB-TGCONSTRCT-120117-1), (Li et al., 2020a), *Tg(rag2:DsRed)* (with lymphocytes labeled, http://zfin.org/ZDB-TGCONSTRCT-131022-4), (Cheng et al., 2020) and *Tg(lck:lck-eGFP)* (with T cells labeled, https://zfin.org/ZDB-TGCONSTRCT-070117-48), (Jerison and Quake, 2020), were purchased from CZRC (China Zebrafish Resource Center, http://en.zfish.cn/) and were maintained according to standard protocols (Renshaw et al., 2006). The transgenic zebrafish used in this study included innate immune cell labeled *Tg(lyz:DsRED2)*; *Tg(mpeg1:EGFP)*, as well as adaptive immune cell labeled *Tg(rag2:DsRed)* or *Tg(lck:lck-eGFP)*. Specifically, since the zebrafish rag2^+^ cells could respond to inflammatory signals (Li et al., 2014) and the rag2^*E450fs*^ mutant even lacked mature T cells (Moore et al., 2016), *Tg(rag2:DsRed)* was used to analyze the lymphocytes’ response with SBMIE modeling. The Institute of Hydrobiology of the Chinese Academy of Sciences approved the use of animals in this study. All experiments followed the committee’s guidelines.

Shaanxi Tengmai Biotechnology Co., Ltd. in China supplied the seabuckthorn fruit powder, which was made using a low-temperature continuous vacuum belt dryer. The frozen fruit was squeezed to extract juice after thawing and was then left to settle. Then the juice, excluding the oil, was concentrated and dried using the low-temperature continuous vacuum belt dryer. After this process, the seabuckthorn fruit powder was obtained. The negative (FM diet) and positive (SBM diet) zebrafish diets were created using a previously published zebrafish SBMIE modeling formula (Bravo-Tello et al., 2017). To test the modifying effect of seabuckthorn as an effective additive, 5,000 ppm of seabuckthorn was added to the positive control (SBM diet) in the diet formulation. A previously published method was used to conduct a feeding trial in adult fish to model soybean-induced enteritis (SBMIE) with modifications (Wu et al., 2020). In brief, 270 3-month old wide type zebrafish (with the initial weight 0.175 ± 0.05 g) were fed the FM diet for 2 weeks. Then, 6-week feeding trials of experimental diets, including FM (negative control), SBM (positive control), as well as the drug (seabuckthorn) groups (SBM +35ppm seabuckthorn fruit powder) (Table 1), were carried through feeding twice a day. The feeding trials for the SBMIE larvae model followed our recently published protocol (Xie et al., 2021). In brief, a 4-day feeding trial of experimental diets from 5 to 8 dpf using larvae of *Tg(lyz:DsRED2); Tg(mpeg1:EGFP)* was completed for examining the response of innate immune cells. A 10-day feeding trial of experimental diets from 17 to 27 dpf using *Tg(rag2:DsRed)* or *Tg(lck:lck-eGFP)* larvae was completed for adaptive immune cells.

**TABLE 1:** Formulation of experimental diets.

Raw material g/kg	FM	SBM	SB
Fish meal	555	250	250
Soybean meal	0	500	500
Wheat meal	255	110	110
Starch	50	50	50
Fish oil^a^	30	60	60
Mineral premix^b^	10	10	10
Vitamin premix	10	10	10
Seabuckthorn	0	0	5
Cellulose	90	10	5
Gross weight(g)	1000	1000	1000

a Per kilogram of mineral premix (g kg^−1^): MnSO_4_·H_2_O (318 g kg^−1^Mn), 1.640 g; MgSO_4_·H_2_O (150 g kg^−1^ Mg), 60.530 g; FeSO_4_·H_2_O (300 g kg^−1^ Fe), 23.110 g; ZnSO_4_·H_2_O (345 g kg^−1^ Zn), 0.620 g; CuSO_4_·5H_2_O (250 g kg^−1^ Cu), 0.010 g; KI (38 g kg^−1^ I), 0.070 g; NaSeO_3_ (10 g kg^−1^ Se), 0.005 g. All ingredients were diluted with cornstarch to 1 kg.

b Per kilogram of vitamin premix (g kg^−1^): retinyl acetate (500,000 IU g^−1^), 2.40 g; cholecalciferol (500,000 IU g^−1^), 0.40 g; DL-a-tocopherol acetate (500 g kg^−1^), 12.55 g; menadione (230 g kg^−1^), 0.80 g; cyanocobalamin (10 g kg^−1^), 0.83 g; D-biotin (20 g kg^−1^), 4.91 g; folic acid (960 g kg^−1^), 0.40 g; thiamin hydrochloride (980 g kg^−1^), 0.05 g; ascorhyl acetate (930 g kg^−1^), 7.16 g; niacin (990 g kg^−1^), 2.24 g; meso-inositol (990 g kg^−1^), 19.39 g; calcium-D-pantothenate (980 g kg^−1^), 2.89 g; riboflavin (800 g kg^−1^), 0.55 g; pyridoxine (980 g kg^−1^), 0.59 g. All ingredients were diluted with corn starch to 1 kg.

### Sampling

Adult zebrafish hindgut, a third of the whole intestine, from bend 2 to the anus (Lickwar et al., 2017), were used for sampling. Tissue samples were collected for quantitative PCR, HE staining, oil-red staining, immunohistochemistry, and transcriptome analysis (Wu et al., 2018). We used the hindgut tissue from both female and male fish for the omics investigation to prevent sex-biased differences. The complete hindgut contents were collected in sterile tubes and preserved at −80°C for 16S rRNA gene sequencing (Deng et al., 2020). For the sampling of larvae, only the caudal part of 27 dpf larvae has been sampled for HE (Hematoxylin–eosin) staining, during the imaging of the adaptive immune cells.

### Evaluation of Growth Performance

To evaluate growth performance, the 3 months old zebrafish of wide type used for SBMIE modeling were randomly distributed and fed in 9 tanks. For every group, there were three repetitions and 30 fish in each tank. In the tank, the water temperature was 28 ± 0.5°C, and the pH was 7.2–7.6. Meanwhile, DO (dissolved oxygen) was kept above 5.0 mg/L. To obtain more accurate growth parameters, zebrafish were fed the experimental diet at a rate of 3% of their body weight per meal, with two meals per day and a half-hour satiety interval. Each meal was fed 6 times, with the dosage gradually decreasing to ensure that the feed for each meal was consumed by the experimental fish. During this process, we discovered that zebrafish could consume the feed without any waste.

As most of the ordinary parameters of growth performance were found to not be significantly changed (data not shown), a 3D micro-CT (Bruker SkyScan 1276 Micro-CT Scanner) was used to measure the volume of total adipocyte tissue in the zebrafish just after the feeding trial using the previously described method (Hasumura et al., 2012; Zang et al., 2018). To prevent motion interference, the zebrafish were anesthetized with tricaine methanesulfonate (MS222, Sigma-Aldrich, USA), before being put into the scanner (Xiong et al., 2017). Insta-Recon software was used to reconstruct and visualize the fish images, and CT analyzer software was used to analyze areas of fish adipose tissues and their entire trunk (Ondruš et al., 2021).

### Histological and Immunohistochemistry Analyses

For all the histological and immunostaining analyses, the tested hindgut tissues were from three fish and there were at least 3 slice repetitions for the tissue from each fish. HE staining was used to show the structure of the mucosal fold in the hindgut (in both adult zebrafish and 27 dpf larvae) as well as the reticuloendothelial structure in the liver to demonstrate basic pathology (in adult zebrafish). According to previously published methods, oil-red staining was used to visualize the oil droplets in the liver (Wu et al., 2018). For both HE and oil-red staining analysis, the last third of the intestinal tissues from three fish in each group were used to make sections (10 μm), and at least ten sections per fish were observed in a bright field under a microscope. To observe the adult zebrafish intestine and liver tissue, a microscope (Olympus, BX53) with a magnification of 20-fold was used. Meanwhile, for the larva tissue, the Aperio VERSA Slide Scanner (Aperio VERSA 8, Leica) with a magnification of 40-fold was applied. The zebrafish anti-CD4-1 antibody (GeneTex, USA, GTX16589) was used in immunohistochemistry analysis to label mainly T helper cells in the gut and liver to further illustrate the inflammation at the lymphocyte level, as previously described (Wu et al., 2018). DAPI was also used to stain the nuclei in parallel.

### RNA Extraction and qPCR Validation

The RNA extraction and qPCR analysis were carried out following the procedures outlined in our previously published paper (Wu et al., 2018; Wu et al., 2020). All RNA extractions were performed immediately following the sampling of either the hindgut or liver tissues or at the same time as the sampling for the transcriptomic study. Supplementary Table S1 lists the primers used to quantify intestinal or hepatic DEGs. The tested hindgut or liver tissues for qPCR analysis were from 6 fish which contained 3 repetitions. In each repetition, hindgut or liver tissues from a female fish and a male fish were mixed.

### Library Preparation and Sequencing for Transcriptomic Data

Transcriptomic analysis was used to determine the gene expression profile of distal intestine and hepatic RNA (*n* = 3) in adult fish from all groups, including the negative control (FM group), the positive control (SBM group), and the tested drug inclusion group (SB group). The process for preparing the gene library and sequencing the transcriptome was consistent with previously described methods (Johnson and Hofmann, 2016). To summarize, sequencing libraries were prepared using the NEBNext RUltraTM RNA Library Prep Kit for Illumina R (NEB, USA) according to the manufacturer’s guidelines, and the quality of the libraries was determined using the Agilent Bioanalyzer 2100 system. On an Illumina platform (NovaSeq 6000), the library preparations were sequenced and 150 bp paired-end reads were produced.

### Transcriptome Assembly, DEG Analysis and Functional Annotation

Hisat2 (version 2.0.4) (Kim et al., 2019) was used to map the clean reads to the zebrafish genome (GRCz11, https://www.ncbi.nlm.nih.gov/genome/?term=txid7955[orgn]). The number of reads that were mapped were counted verse by verse (version 0.1.5). The reads count was imputed into DESeq2 (version 1.24.0) to analyze DEGs. To select DEGs for analysis, DESeq2 (Love et al., 2014) was used with | log2FoldChange | > 1 and padj 0.05. For annotation, clusterProfiler (version 3.12.0) (Yu et al., 2012) was used to perform enrichment analysis of both GO (Gene Ontology) terms and KEGG (Kyoto Encyclopedia of Genes and Genomes) pathways, with *p* < 0.05 considered significant enrichment. When the parameters used were not listed, default parameters were used.

### 16S rRNA Sequencing of Intestinal Microbiota

The composition of bacterioplankton was analyzed using NovaSeq sequencing of 16S rRNA gene amplicons (*n* = 6) as previously published protocol (Xie et al., 2021). In brief, the V3V4 regions of the 16S rRNA gene were amplified. After construction of the libraries, the paired-end 250-nucleotide reads were obtained using the Illumina NovaSeq platform. Using the DADA2 plugin, raw sequences were trimmed, quality filtered, denoised, merged, chimera, and dereplicated (Callahan et al., 2016). Reads with 100 percent nucleotide sequence identity across all samples were assigned to operational taxonomic units (OTUs), and taxonomy was assigned to Non-singleton amplicon sequence variants (ASVs) using the classify-sklearn nave Bayes taxonomy classifier in the feature-classifier plugin (Bokulich et al., 2018) against the Greengenes reference database (DeSantis et al., 2006).

### *In Vivo* Imaging of Immune Cells in Zebrafish Larvae

Following previously published methods for zebrafish larvae imaging (Bravo-Tello et al., 2017; Coronado et al., 2019; Ikeda-Ohtsubo et al., 2020), *in vivo* imaging of intestinal immune cells in zebrafish was conducted according to our recently published protocol (Li et al., 2021; Xie et al., 2021). As the innate cell’s response could be examined at a very early stage, the feeding trial was done from 5 dpf to 9 dpf, using the neutrophil and macrophage labeled Tg lines. At an adaptive aspect, soybean meal was fed after the grass carp’s body length was at least 10 cm making it a juvenile in aquaculture. The feeding protocol for live imaging of the lymphocytes was to mimic the commercial fish’s feeding strategy. Zebrafish intestinal adaptive immune function has been well developed after 18 dpf (the starting time point for feeding trial to test lymphocytes’ response) and up to 27 dpf when it is nearly an adult fish (the ending time point). Thus, the SBMIE modeling used to analyze the lymphocytes in the lymphocyte labeled larva was carried out in well-developed hindguts, allowing us to test the possibility of seabuckthorn mitigating the side effect of soybean meal diet on adaptive immunity.

Methodologically, before the feeding trial in larvae, to prepare the experimental feeds, the diets for the FM, SBM, and SB groups were crushed and passed through an 80-mesh sieve. 200 larvae were used for each group to analyze either innate or adaptive immune cells. The water temperature was kept at 28 ± 0.5°C. For each group, from 0 to 3 dpf, the larvae were kept in Danieau’s solution (http://cshprotocols.cshlp.org/content/2013/5/pdb.rec074260.full) with the anti-fungal agent methylene blue (MB, 0.0001%–0.0005%), later the larvae were kept in Danieau’s solution. During the feeding trial to analyze either innate or adaptive immune cells, larvae were fed three times per day. On the day for imaging, larvae were anesthetized with MS222 before being embedded in 1% LMP Agarose (Invitrogen, dissolved in Danieau’s solution). A Leica SP8 microscope was used to examine the experimental larvae. At least 10 larvae were observed under a microscope for each group. To generate a picture for the entire posterior part of the intestine, a composition of several stacks (*n* = 13) was merged for imaging the hindgut signals.

The feeding protocol is shown in Supplementary Figure S1. To analyze innate immune cells, between 5 and 8 dpf, the larvae of *Tg(lyz:DsRED2); Tg(mpeg1:EGFP)* were kept in Danieau’s solution and fed experimental diets. Upon imaging of innate immune cells, including neutrophils (lyz:DsRED2 labeled) and macrophages (mpeg1:EGFP labeled), was performed in the hindgut (posterior part) of the larvae of *Tg(lyz:DsRED2); Tg(mpeg1:EGFP)* at 9 dpf (Xie et al., 2021). Meanwhile, to test the cellular response of adaptive immune cells, the immature (rag2:DsRed labeled) (Zhang et al., 2018) and mature lymphocytes (lck-eGFP labeled) (Langenau and Zon, 2005), in the intestine (posterior part) of *Tg(rag2:DsRed)* and *Tg(lck:lck-eGFP)* larvae (Xie et al., 2021), were imaged. After 12 days (5–16 dpf) feeding of Larval AP100 Diet (Zeigler) as well as 10 days of experimental diets feeding from 17 dpf, the intestinal lymphocyte in larvae were imaged at 27 dpf.

### Statistical Analysis

The FIJI/ImageJ software (Póvoa et al., 2021) was used to calculate the area and intensity of HE stained mucosal folds, oil drops, fluorescent cells, and immunohistochemistry signals. In the oil-red staining result for hepatic images, the area ratio of oil drops vs. total area was then calculated. During the immunohistochemistry analysis, the ratio of CD4^+^ cells vs. total DAPI-stained nuclei was calculated to reveal the hepatic pathology. The fluorescent-labeled signals or immunostained signals for intestinal signals were calculated using previously published methods (Xie et al., 2021). Data were analyzed in EXCEL for the correlation analysis comparing fold changes between qPCR and RNA-Seq results. The R-value was calculated using Pearson’s correlation. The information was processed by the GraphPad Prism 5 software (GraphPad Software Inc. CA, United States). The bar diagram represented mean ± SEM. For the comparison between two groups, data were analyzed using a T-test. While for the comparison among multiple groups, data were analyzed using one-way ANOVA followed by a Duncan’s test. Differences were considered significant at *p* < 0.05 (*), *p* < 0.01 (**) and *p* < 0.001 (***).

## Results

### Prediction of Seabuckthorn and Its Effective Components to Treat Fish SBMIE

GeneRank selected the key 300 SBMIE DEGs based on SBMIE-related DEGs in grass carp and the protein-protein interaction network (Supplementary Table S2). Then, using the components targeting those key genes, a medicine containing effective components was chosen from TCMID. The results revealed that seabuckthorn contained five effective components, as well as the component’s key regulated genes (Table 2), based on TCMID correlations between drug components and responsive genes (Huang et al., 2018). Meanwhile, 33 DEGs from the transcriptome of grass carp with SBMIE were matched to the effective components (Table 3), potentially narrowing the drug range to seabuckthorn. As a result, seabuckthorn was chosen as a candidate to treat foodborne enteritis in fish.

**TABLE 2:** Predicted effective components and target genes from TCMID database.

Drug name	Effective components	Target genes
Seabuckthorn	Capric acid	*rspo4*, *pcsk1*
	Caproic acid	*plg*, c*asr*
	Caprylic acid	*Acacb*
	Gallic acid	*jun*, *mapk3*, *p53*, *mapk1*
	Malic acid	*Acacb*

**TABLE 3:** The seabuckthorn targeted DEGs in grass carp suffering SBMIE.

ID	Gene	Annotation
CI01000346_00033095_00038432	*p53*	tumor protein p53
CI01000071_04534337_04569325	*mapk1*	mitogen-activated protein kinase 1
CI01000026_10831971_10837028	*f2*	coagulation factor II, thrombin
CI01000004_11432849_11451784	*yes1*	proto-oncogene 1, Src family tyrosine kinase
CI01000010_02553536_02561950	*zap70*	zeta-chain (TCR) associated protein kinase 70 kDa
CI01000340_15454365_15456711	*rspo4*	R-spondin 4
CI01000010_05594817_05606739	*abl2*	proto-oncogene 2, non-receptor tyrosine kinase
CI01000325_02484476_02491983	*kdr*	kinase insert domain receptor
CI01000071_01149617_01177048	*acacb*	acetyl-CoA carboxylase beta
CI01000029_03698769_03707177	*plg*	plasminogen
CI01000059_06430220_06439547	*casr*	calcium sensing receptor
CI01000037_04313204_04343186	*gsk3b*	glycogen synthase kinase 3 beta
CI01000301_00660823_00677721	*gsk3b*	glycogen synthase kinase 3 beta
CI01000152_02905331_03070904	*ephb1*	EPH receptor B1
CI01100888_00000121_00001066	*kdr*	kinase insert domain receptor
CI01000339_02221790_02222160	*p53*	tumor protein p53
CI01000004_09490844_09498265	*lyn*	proto-oncogene, Src family tyrosine kinase
CI01022064_00000512_00000912	*cdh3*	cadherin 3
CI01000325_05045369_05061497	*esr1*	estrogen receptor 1
CI01000029_03746973_03752660	*plg*	plasminogen
CI01000001_00580789_00602408	*src*	proto-oncogene, non-receptor tyrosine kinase
CI01022123_00000351_00000764	*cdh3*	cadherin 3
CI01000054_01634335_01637623	*rdh13*	retinol dehydrogenase 13
CI01000051_03431193_03432454	*jun*	proto-oncogene, AP-1 transcription factor subunit
CI01072909_00000766_00002054	*pcsk1*	proprotein convertase subtilisin/kexin type 1
CI01000116_00002666_00003048	*spink1*	serine peptidase inhibitor, Kazal type 1
CI01000006_12380830_12394437	*pcsk1*	proprotein convertase subtilisin/kexin type 1
CI01000053_02118918_02119910	*mos*	Moloney murine sarcoma viral oncogene
CI01000339_02215633_02219758	*p53*	tumor protein p53
CI01000016_02328065_02334778	*rdh13*	retinol dehydrogenase 13
CI01022074_00000258_00000685	*cdh3*	cadherin 3
CI01000069_00059923_00065619	*mapk3*	mitogen-activated protein kinase 3
CI01000059_06430220_06439547	*casr*	calcium sensing receptor

### Effect of Seabuckthorn on Gut-Liver Pathology in SBMIE Adult Model

**FIGURE 1:**
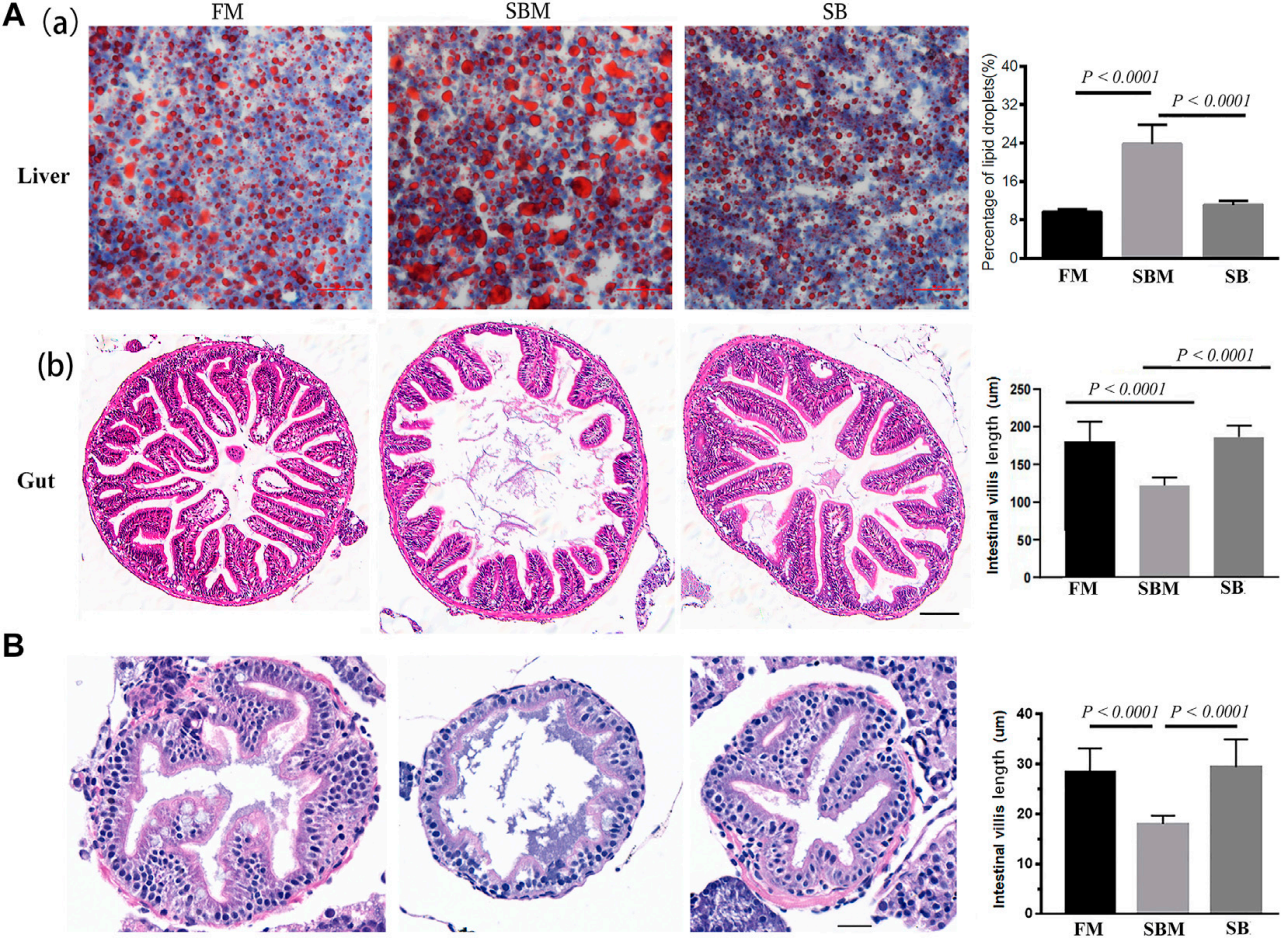
Histological analysis of seabuckthorn’s effect pathologically. **(A)** In the SBMIE adult model, both decreased hepatic oil-red stained droplets **(a)** and increased length of intestinal villi **(b)** were found relieved in wide type zebrafish from the SB group compared to the SBM group. The scale bar in hepatic and intestinal images of adult zebrafish indicated 50 μm. **(B)** HE staining of intestinal mucosa as well as quantitative analysis of intestinal villi length in 27 dpf wide type larva feed FM, SBM and SB diets. Scale bar: 20 μm. FM: fish meal diet; SBM: soybean meal diet; SB: seabuckthorn supplementary SBM diet. All bar diagrams of quantitative analysis for hepatic oil red-stained signals or the intestinal villi length were at the right side of the typical images.

When the SBM group (positive control) was compared to the FM group (negative control), HE staining revealed an intestinal mucosa lesion (Figure 1) and loosening of the hepatic reticuloendothelial structure (Supplemntary Figure S2) (negative control). The inclusion of seabuckthorn at a concentration of 35 ppm significantly reduced the appearance of such pathological change in the SBMIE adult model, as evidenced by a very significantly (*p* < 0.0001) decreased percentage of oil red-stained droplets (Figures 1A,a) and a more compact reticuloendothelial structure (Supplementary Figure S2) in the liver, as well as very significantly (*p* < 0.0001) shortened villi (Figures 1A,b). Both the amount and particle size of inflammation-related lipid droplets in the SBM group were reduced a lot in the SB group (Figures 1A,b). Additionally, in the SBMIE larvae model, the intestinal villi in 27 dpf larvae from the SB group were still within the normal length comparable to that in the FM group. However, in the SBM group the intestinal fold had significantly (*p* < 0.0001) shortened villi length in the SBM group (Figure 1B). Longer intestinal villi (*p* < 0.0001) were seen in the SB group compared to the SBM group at 27 dpf in the posterior part of the intestine (Figure 1B). Yet, the villi structure in the last portion of the hindgut (near the anus) did not differ much in length (Supplementary Figure S3).

**FIGURE 2:**
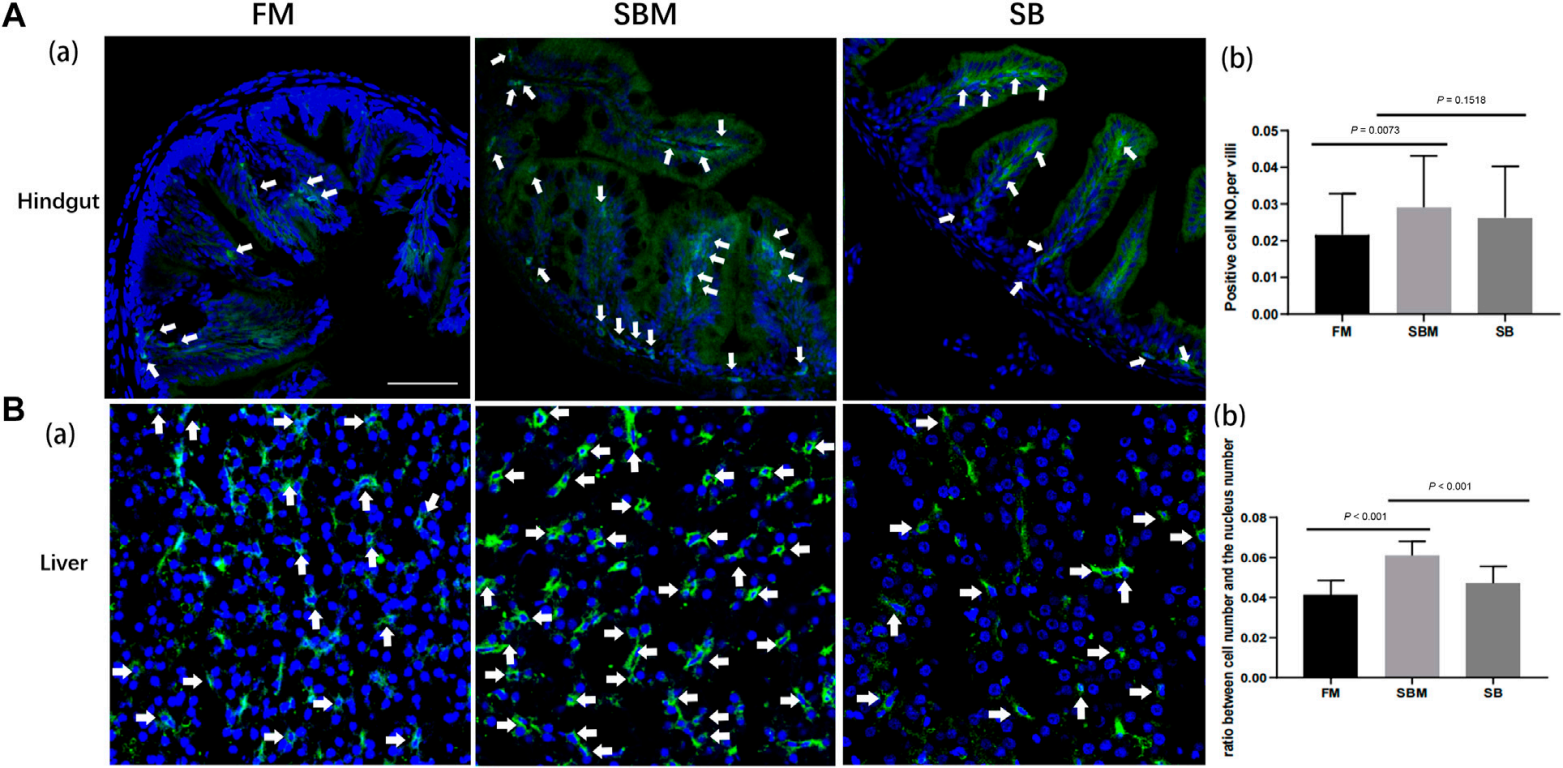
The effect of seabuckthorn on intestinal and hepatic T helper cells reflected by immunohistochemistry signals. **(A)** The representative images **(a)** and the bar diagram for quantitative analysis **(b)** of intestinal CD4 labeled Th cell signals as well as the quantitative analysis of signals in FM, SBM and SB groups; **(B)** The representative images **(a)** and the bar diagram for quantitative analysis **(b)** of hepatic CD4 labeled Th cell signals as well as the quantitative analysis of signals in FM, SBM and SB groups. The typical signals were indicated by arrows. Scale bar: 100 μm.

The immunohistochemistry result for the CD4 protein indicated the presence of the intestinal T helper lymphocyte. This was reflected by the CD4 signals that were detected in the intestinal villi only in the LP layer, with very few at the base of the mucosal fold (Figure 2A). Quantitative analysis of the number of CD4^+^ cells divided by the length of intestinal villi revealed no significant difference in the ratio between the SB and SBM groups (data not shown). The SB group had a lower ratio of hepatic CD4^+^ cells (Figure 2B) compared to the SBM group. Quantitative analysis of signals revealed a significant decrease in the ratio of hepatic CD4^+^ cells to total cells, as indicated by the DAPI stained nucleus (Figure 2B). Additionally, it is interesting to note that some hepatic CD4^+^ cells had prolongations.

### Effect of Seabuckthorn on Body Fat in SBMIE Adult Model

**FIGURE 3:**
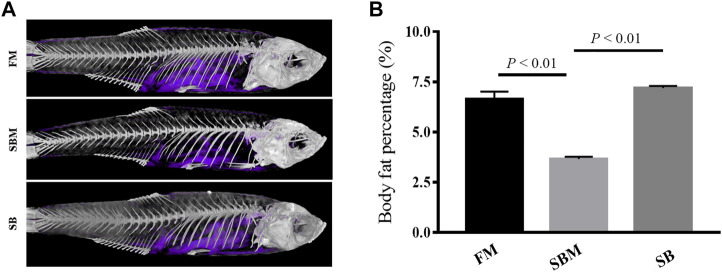
The reconstructed image of body fat revealed by 3D micro-CT in adult zebrafish from FM, SBM and SB groups. The body fat in the fish trunk was colored purple. **(A)** The images of body fat (in purple) within the fish from all groups; **(B)** The bar diagram to show percentages of body fat in whole fish trunk.

A 3D micro-CT scanner was used to determine body fat. After scanning anesthetic adult zebrafish, the image data was processed to create a 3D structural reconstruction and quantitatively analyzed. The results indicate that the percentages of body fat in the FM, SBM, and SB groups were 8.99 percent, 2.14%, and 6.83%, respectively. The reconstructed three-dimensional images may correspond to areas of body fat in zebrafish. The area of body fat in SBM fish was significantly less than that in FM and SB fish, whereas the area of body fat in SB fish appeared to be comparable to that in FM fish (Figure 3).

### Enriched GO Terms and KEGG Pathways of Intestinal and Hepatic DEGs

**FIGURE 4:**
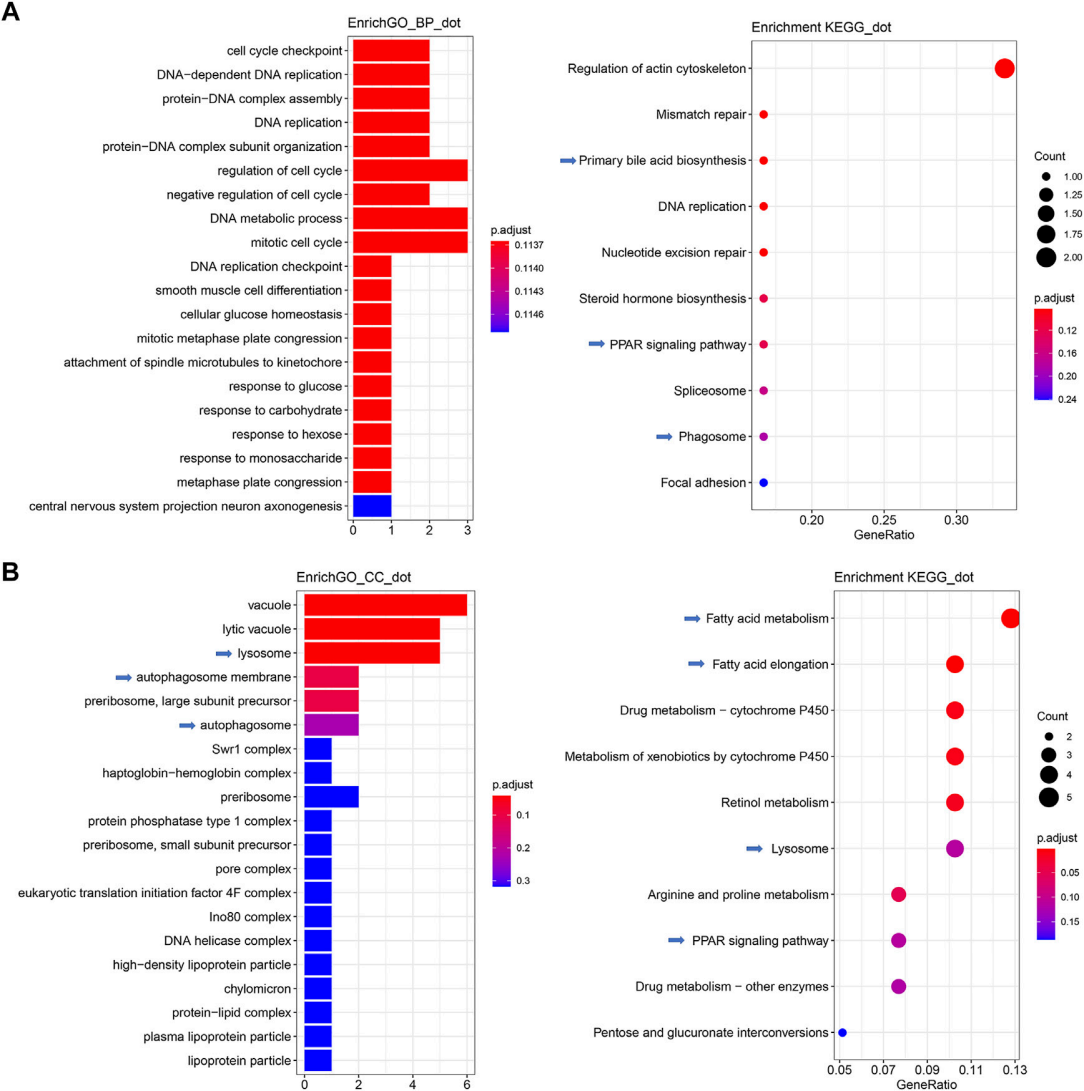
GO terms and KEGG pathways enriched for both intestinal and hepatic DEGs in comparison between SB and SBM groups in zebrafish SBMIE adult model. **(A)** Key intestinal terms biological processes (left) and KEGG pathways (right); **(B)** Key hepatic terms for cellular components (left) and KEGG pathways (right). The immune-related terms and pathways are labeled with arrows.

The qPCR validation result (Supplementary Table S1) demonstrated that the fold-changes between DGE and qPCR were highly correlated (R > 0.8) across 22 reactions encoding 9 genes, indicating that the current transcriptomic study successfully decoded gene expression. The results of the comparison between the SBM and FM groups (Supplementary Figure S4) revealed intestinal GO terms associated with immune responses, such as “humoral immune response” and “complement activation.” Between the SBM and FM groups, the KEGG pathways “lysosome,” “cytokine-cytokine receptor interaction”, and “ferroptosis” were identified for the hepatic inflammation, while the lipid metabolism-related pathways “PPAR signaling pathways” and “fatty acid elongation” were also enriched. This resulted from the successful transcriptomic modeling of SBMIE. Then, using intestinal DEGs, the drug-incorporated group could be analyzed for its moderating effect on SBMIE (Supplementary Table S3). Numerous terms related to cell proliferation were included in the enriched GO terms for biological processes, including “cell cycle checkpoint,” “DNA-dependent DNA replication,” “protein-DNA complex assembly”, “DNA replication,” “protein-DNA complex subunit organization,” “cell cycle regulation,” “negative regulation of cell cycle,” “DNA metabolic process,” “mitotic cell cycle,” “DNA replication checkpoint,” “mitotic metaphase plate congression,” and “attachment of spindle” (Figure 4A, left; Supplementary Table S4). In addition to the enriched GO terms, the enriched KEGG pathways (Figure 4, right; Supplementary Table S5) included cell division-related pathways such as “regulation of actin cytoskeleton,” “mismatch repair,” “DNA replication,” “nucleotide excision repair,” and “splice some.” Additionally, the pathways “primary bile acid biosynthesis,” “PPAR signaling pathway,” and “phagosome” were also enriched for intestinal DEGs.

Meanwhile, for hepatic DEGs derived from the comparison of the SB vs. SBM groups (Supplementary Table S6), enriched GO terms for cellular components included terms, such as “lysosome,” “autophagosome membrane,” “autophagosome,” “high-density lipoprotein particle,” “protein-lipid complex,” “plasma lipoprotein particle,” and “lipoprotein” (Figure 4B, left; Supplementary Table S7). Additionally, the hepatic enrichment analysis (Figure 4B, right; Supplementary Table S8) also revealed the GO terms “Lysosome” and “PPAR signaling pathway” as well as KEGG pathways “fatty acid metabolism” and “fatty acid elongation.”

The raw data from the current transcriptome sequencing analysis has been submitted to the Genome Sequence Archive (GSA) database (http://gsa.big.ac.cn/index.jsp) under the BioProject identifier < PRJCA005917> and data ID < CRA004582>. Within this BioProject identifier, there was one data ID for the transcriptomic data and another for the 16s rRNA gene sequencing result, as described below. This study shared the same positive (SBM group) and negative (FM group) controls with a published study on sinomenine’s anti-enteritis effect (Xie et al., 2021), since both drugs were analyzed for the anti-SBMIE effect at the same time using the same batch of fish.

### Effect of Seabuckthorn on Microbiota OTU and Taxa Composition

**FIGURE 5:**
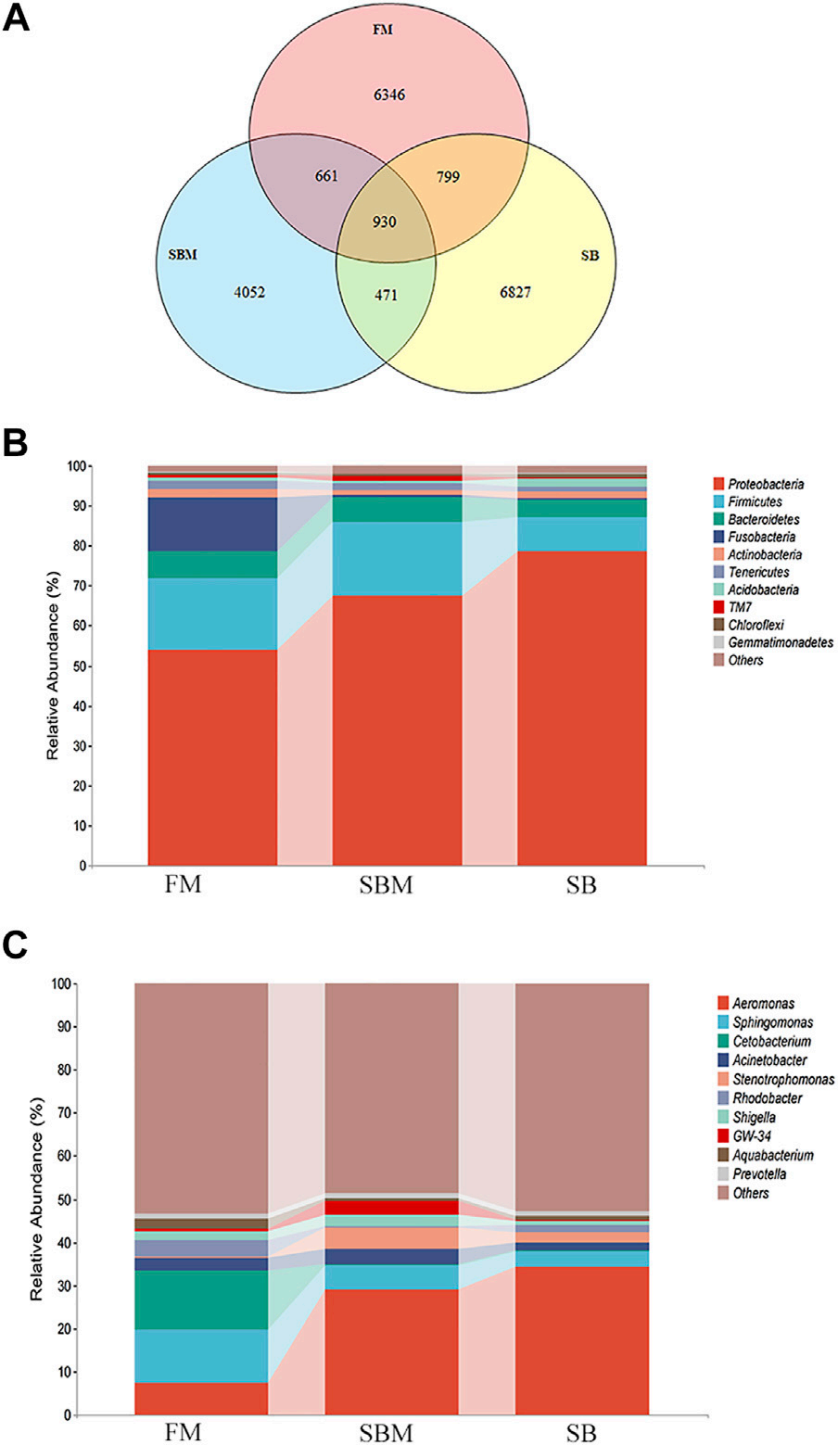
Analysis of intestinal microbiota OTU and composition influenced by seabuckthorn dietary inclusion in zebrafish SBMIE adult model. **(A)** Venn diagram of OTU in FM, SBM and SB groups; **(B)** hindgut bacteria composition at phylum level; **(C)** hindgut bacteria composition at the genus level. The alteration trends between adjacent bars for the current revealed taxa were indicated by the interval belts.

There were 8434, 5976, and 9027 OTUs in the hindgut of adult fish belonging to the FM, SBM, and SB groups, respectively. As illustrated in the Venn diagram, 799 OTUs overlapped between the SB and FM groups, but only 471 OTUs overlapped between the SB and SBM groups (Figure 5A). As illustrated in Figure 5A, the overlap between the seabuckthorn and FM groups covered 799 OTU, while the overlap between the seabuckthorn and SBM groups contained 471 OTU. The SB group-specific region contained 6827 OTU, which was slightly more than the FM group-specific region’s 6346 OTU and significantly more than the SBM group-specific region’s 4052 OTU. The OTUs identified a large number of phyla in the fish intestinal microbiota to estimate their abundance.

Among the 10 phyla with the most abundance, compared to the SBM groups, in the SB group, the increased phyla included Proteobacteria, Actinobacteria, Acidobacteria, Chloroflexi, and Gemmatimonadetes, while decreased phyla included Bacteroidetes, Firmicutes, Fusobacteria, Tenericutes and TM7 (Figure 5B). At the genus level (Figure 5C), among the most abundant 10 genera, compared to the SBM groups, in the SB group, the increased genera included *Aeromonas*, *Rhodobacter*, *Pelomonas* and *Aquabacterium*, while decreased genera included *Sphingomonas*, *Cetobacterium*, *Acinetobacter* and *Shigella*. More specifically, the relative abundance of the 16S RNA gene was listed for each sample at both phylum and genus levels in Supplementary Table S9.

The raw data of the current 16S rRNA gene sequencing analysis was submitted to the Genome Sequence Archive (GSA) database (http://gsa.big.ac.cn/index.jsp) with the BioProject identifier < PRJCA005917> and data ID < CRA004611>. This study shared the same positive (SBM group) and negative (FM group) controls with the parallel analysis on the sinomenine test since seabuckthorn and sinomenine were analyzed for the anti-SBMIE effect at the same time using the same batch of fish.

### Effect of Seabuckthorn on Intestinal Immune Cell of Larvae

**FIGURE 6:**
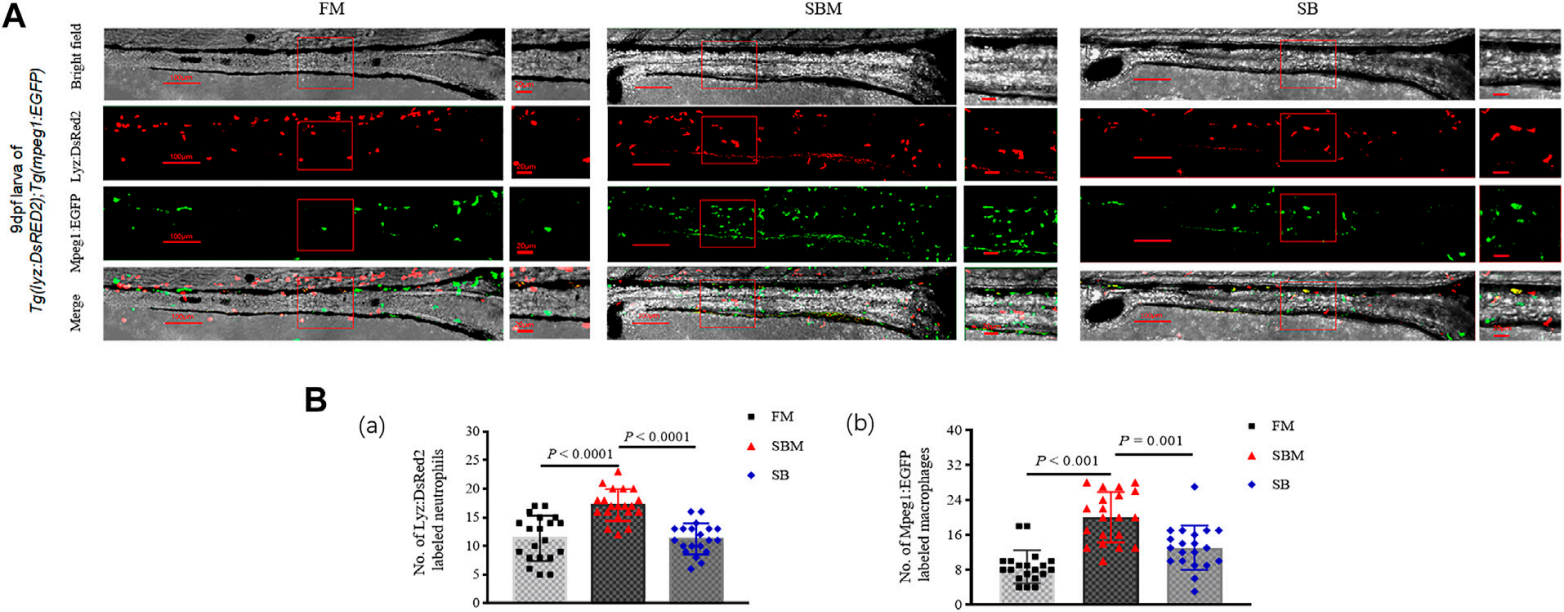
Imaging of innate immune cells and expressional analysis of immune-related genes in zebrafish SBMIE larva model. **(A)** lyz:DsRED2 labeled neutrophils and mpeg1:EGFP labeled macrophages imaging in the intestine (posterior part, *n* = 10) using 9 dpf larvae of *Tg(lyz:DsRED2)*; *Tg(mpeg1:EGFP)*. The square indicated the zoomed in region. The scale bar in whole pictures was 100 μm, while the scale bar in the enlarged view was 20 μm. **(B)** quantitative analysis of the signals using bar-diagrams. **(a)** Number of Lyz:DsRed2 labeled neutrophils in larvae from either FM, SBM, or SB groups; **(b)** No. of Mpeg1:EGFP labeled macrophages in larvae from either FM, SBM, or SB groups. The very significant differences (*p* < 0.01) of both Lyz:DsRed2 and Mpeg1:EGFP labeled cells between SB and SBM groups were indicated by *p-*value.

**FIGURE 7:**
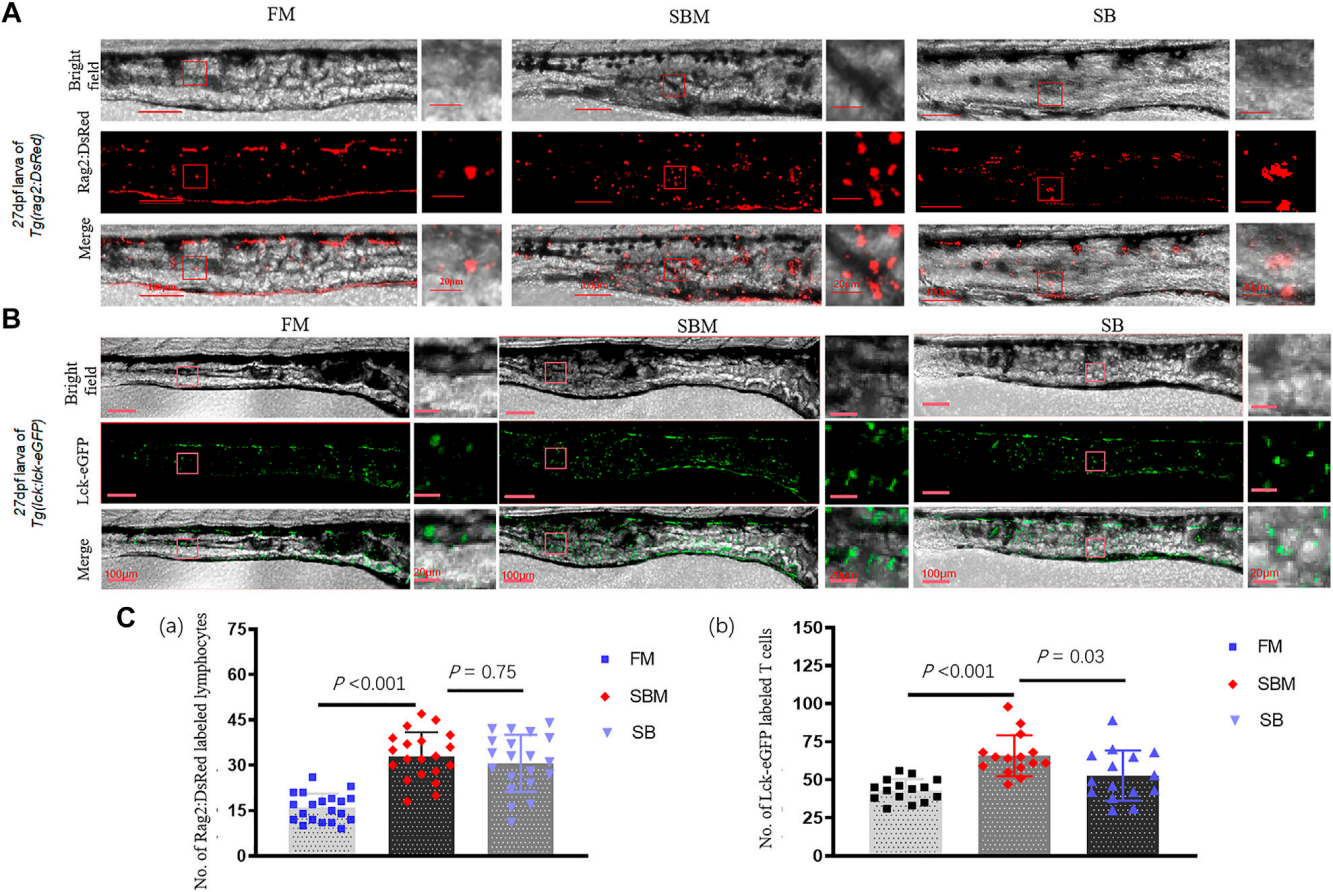
Imaging of lymphocytes and expressional analysis of immune-related genes in SBMIE larvae model. **(A)** rag2:DsRed labeled immature lymphocytes imaging in 27 dpf *Tg(rag2:DsRed)* larvae’s intestine (posterior part, *n* = 10); **(B)** lck-eGFP labeled mature T cells imaging in 27 dpf *Tg(lck:lck-eGFP)* larvae’s intestine (posterior part). The square indicated the zoomed in region. The scale bar in whole pictures was 100 μm, while the scale bar in the enlarged view was 20 μm. **(C)** quantitative analysis of the signals using bar-diagrams. **(a)** Number of Rag2:DsRed labeled lymphocytes in larvae from either FM, SBM, or SB groups; **(b)** Number of Lck-eGFP labeled T cells in larvae from either FM, SBM, or SB groups. The significant differences (*p* < 0.05) of Lck^+^ T cells between SB and SBM groups were indicated by the *p-*value.

At a cellular level, compared to the SBM group (positive control), seabuckthorn could significantly (*p* < 0.01) relieve inflammatory aggregation of innate immune cells, including lyz:DsRED2 labeled neutrophils and mpeg1:EGFP labeled macrophages (Figure 6; Supplementary Figure S5), whilst significantly (*p =* 0.03) reduced inflammatory aggregation of lck^+^ T cells (Figure 7; Supplementary Figure S5) in the intestine (posterior part) of zebrafish larvae in SB group. Morphologically, seabuckthorn could inhibit the formation of protrusions in macrophages in the hindgut (Figure 6). While, compared to the FM group (negative control), there were no significant changes in both number and morphology in the SB group for most observed immune cells, the differences of both number and morphology for rag2:DsRed labeled lymphocytes were observed in Figure 7. The factor that rag2:DsRed labeled lymphocytes in the SB group aggregated in the hindgut and still had cellular protrusions, was similar (*p* = 0.75) to that in the SBM group.

## Discussion

Through molecular, cellular, and bio-informational approaches, this comprehensive characterization revealed the effects of dietary inclusion of seabuckthorn on fish gut-liver immunity as well as intestinal bacterial communities in the zebrafish SBMIE model. This has provided clues for developing effective herb-based feed additives to prevent foodborne disease in aquaculture.

Based on the successful modeling of SBMIE in either adult zebrafish or zebrafish larvae (Xie et al., 2021), we modeled in our positive control both the typical intestinal pathology and the typical inflammatory hepatic lipid deposition, similar to the gut-liver symptoms revealed in carp (Wu et al., 2018) (SBM group). The fact that the outcome of recovered body fat trunk in adult zebrafish from the SB group suggested a positive effect of seabuckthorn’s inclusion in alleviating SBMIE-related symptoms. The pathological data hinted that the SB group had less intestinal and hepatic inflammation. Seabuckthorn’s anti-inflammatory role may be reflected in both the liver and the gut, with fewer hepatic oil droplets and a more compact reticuloendothelial structure, as well as longer intestinal villi. Among the seabuckthorn’s target genes, *mapk1* and *mapk3* may reflect cell proliferation (Masselli et al., 2013), *rspo4* might indicate the promotion of WNT signals, which was important for intestinal crypt formation and renewal (Kriz and Korinek, 2018).

In terms of immunity, the fact that the SB group could inhibit both the gathering of innate cells and the formation of protrusions on macrophages was echoed by seabuckthorn’s target gene *plg*. During SBMIE, zebrafish plasminogen may play a role in inflammation-stimulated macrophage recruitment as previously reviewed (Miles et al., 2012). Fewer protrusions may indicate less activation via forming phagocytic synapses (Goodridge et al., 2011; Li et al., 2020c). At an adaptive immunity level, seabuckthorn had less influence on lymphocyte differentiation, as evidenced by unaltered SBM-induced aggregation and protrusions formation of rag2:DsRed labeled lymphocytes as well as an insignificantly altered CD4^+^ cell intestinal location pattern. Nonetheless, the fact that fewer intestinal lck^+^ signals SB target gene *zap70*, a tyrosine kinase important for TCR signaling and late T cell activation (Carpino et al., 2004) suggests a possible inhibition of T cell maturation. However, as demonstrated by the effects on the gut-liver axis, our discovery that the inclusion of seabuckthorn reduced the number of CD4^+^ cells in the liver was consistent with seabuckthorn’s intestinal effect on T cells. In addition, the few hepatic CD4^+^ cells with prolongations might suggest the tissue-resident CD4^+^ monocytes as previously reported in zebrafish (Dee et al., 2016; Lin et al., 2019).

As a result, a systemic study at the omics level made sense to reveal the underlying molecular mechanisms. The DEGs obtained from the comparison of the SB and SBM groups already provided some hints for intestinal or hepatic immune reactions caused by seabuckthorn. For example, increased intestinal expression of *c1qc* may indicate improved complement system function, which may be beneficial to the zebrafish commensal microbiota (López Nadal et al., 2020). The downregulation of *cdt1*, *chaf1b*, *nabp1b*, and other genes in the SB group compared to the SBM group may indicate a reduction in intestinal cell proliferation. A higher level of *apoa4b* in the liver may indicate improved lipid absorption and metabolism (Qu et al., 2019b; Hu, 2020). The effect of SB on lipid deposition was consistent with the target gene acacb of seabuckthorn, which was recently proposed as a therapeutic target for metabolic syndrome (Chen et al., 2019).

By incorporating DEGs into enrichment analysis, the currently revealed GO terms and KEGG pathways, as well as clues for improved intestinal cell proliferation, such as DNA replication and cell cycle regulation, were discovered. As well, the possibility that seabuckthorn’s target gene *p53* may suggest that seabuckthorn supplementation could reduce apoptosis. On the other hand, in terms of immunity, the KEGG pathway “phagosome” was discovered in the comparison of the SB and SBM groups, as phagocyte is an innate immune bioprocess primarily found in fish macrophages. Similar to the SBMIE larvae model, innate immune cells were found to be significantly regulated by SB inclusion in terms of number and cellular protrusions. As a result, seabuckthorn may significantly protect intestinal innate immunity by inhibiting inflammatory aggregation and activation of innate immune cells. Autophagy and lysosome-related GO terms and KEGG pathways in the liver were compared between the SB and SBM groups and demonstrated that the inclusion of seabuckthorn to the SBM diet restored dysregulated hepatic autophagy and lysosome function. The increased lysosome-based autophagy of lipid droplets in the hepatocytes (Schulze et al., 2020) could also explain the SB group’s reduced droplets.

Moreover, several KEGG pathways reflected seabuckthorn’s effect. The intestinal “primary bile acid biosynthesis” may indicate that intestinal epithelial function was regulated and might play a role in anti-inflammation (Pavlidis et al., 2015; Hegyi et al., 2018). Among the biological processes enhanced by seabuckthorn in mouse livers, peroxisome proliferator-activated receptor (PPAR) α and PPAR-γ were significantly increased (Pichiah et al., 2012). In zebrafish, the PPAR pathway was shown to be critical for intestinal homeostasis (Qin et al., 2018). Comparing the regulated PPAR signaling pathways between two control groups (SBM vs. FM), the PPAR signaling pathways revealed by both intestinal and hepatic DEGs may suggest a critical role for PPAR signaling in seabuckthorn’s effect on SBMIE alleviation. Additionally, because L-Arginine has been shown to modulate T cell metabolism (Geiger et al., 2016) and arginine has been shown to protect intestinal health (Zheng et al., 2017), the current study identified the hepatic KEGG pathway “Arginine and proline metabolism,” which may promote gut-liver immunity.

Additionally, as a result of the interaction between the gut microbiota and the liver (Deng et al., 2020), our findings indicated that seabuckthorn supplementation resulted in an increase in OTU numbers and an FM-biased bacterial community in the SB group compared to the SBM group. Taxonomic changes were observed at the phylum level, as Actinobacteria were found to produce active metabolites against pathogenic microorganisms (Jami et al., 2015), and the current study revealed an increase in Actinobacteria, suggesting a protective role for the SB group. When comparing the composition of the intestinal taxa of carnivorous and omnivorous fish, the fact that the SB group had higher levels of Acidobacteria and Chloroflexi may be related to improved protein utilization. This is because Acidobacteria and Chloroflexiact as major components of carnivorous fish’s intestinal microbiota (Wei et al., 2019). Reduced TM7 levels may indicate decreased intestinal inflammation, as intestinal TM7 bacterial phylogenies were previously identified as a pro-inflammatory factor in IBD (inflammatory bowel disease) (Kuehbacher et al., 2008). The levels of Bacteroidetes decreased upon inclusion of seabuckthorn, which may be a sign of recovery from SBMIE. In turbot, a carnivorous fish species, the SBM diet’s intestinal mucosal microbiota was dominated by Bacteroidetes (Li et al., 2019). Reduced *Sphingomonas* was identified as a disease biomarker in the zebrafish (Ma et al., 2020), as was *Shigella*, which was associated with enteritis (human Crohn’s disease) (Zhang et al., 2020c). Both of these observations suggested that the fish probably had recovered from enteritis. Increased levels of the probiotic *Rhodobacter* (Liu et al., 2012; Ye et al., 2019) and the butyrate-producing *Aquabacterium* (Gupta et al., 2019) may act as protective factors.

The effect of seabuckthorn on lipid metabolism and microbiota in zebrafish supported the notion that seabuckthorn could correct the lipid metabolism disease produced by a high-fat diet in mice by altering the gut microbiota (Guo et al., 2020). In the current results, decreased adipose tissue deposition suggested less inflammation in the inner organ. The total body fat in the fish trunk is a measure of growth performance. Thus, with fewer inflammatory droplets in the liver and improved gut shape and microbiota, the fish in the SB group were able to maintain body fat levels comparable to those in the FM group. As the possible effective components, the anticipated acids in Table 2 were consistent with previous findings that unsaturated fatty acids in seabuckthorn could protect against infections, prevent allergies, and reduce inflammation (Zielińska and Nowak, 2017). However, only flavonoid glycosides extracted from seabuckthorn leaves were found to be anti-inflammatory in mice with hepatic steatosis (Kwon et al., 2017). Thus, the delicate mechanisms underlying effective components and the routes implicated remain unknown.

In conclusion, by including seabuckthorn fruit powder in the SBM diet, we were able to alleviate the typical gut and liver pathology associated with SBMIE, via enhancing intestinal immunity and hepatic metabolic adjustments. The involved mechanisms may include inhibition of intestinal cell apoptosis, excessive immune cell aggregation, and microbiota dysbiosis in the gut, together with reduced Th cell aggregation and lipid metabolic burden in the liver. Our findings indicated that dietary seabuckthorn supplementation may protect fish from foodborne gut and liver symptoms.

## Data Availability

The datasets presented in this study can be found in online repositories. The names of the repository/repositories and accession number(s) can be found in the article/Supplementary Material.
