# Supplementary material for: Dietary Inclusion of Seabuckthorn (Hippophae rhamnoides) Mitigates Foodborne Enteritis in Zebrafish Through the Gut-Liver Immune Axis
Source: Front Physiol. 2022 Apr 6;13:831226. doi: 10.3389/fphys.2022.831226 (PMC9019508; doi:10.3389/fphys.2022.831226)
Supplement: Supplementary file 5 [file DataSheet1.PDF]

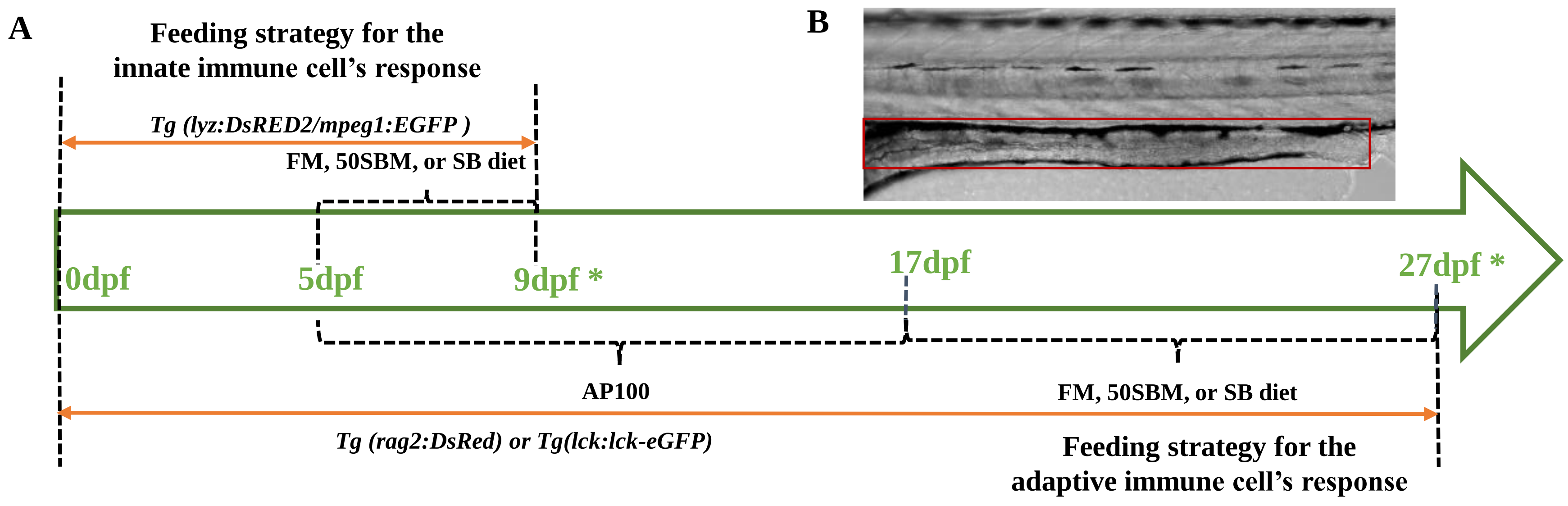

Figure S1 Feeding strategy for analyzing both the innate and adaptive immune cell's response (A) The innate immune cell's response was stimulated by feeding experimental diets from 5dpf to 9dpf. Meanwhile, the adaptive immune cell's response was stimulated by feeding experimental diets from 17 to 27dpf. In addition, before the experimental period, the larvae used to examine the adaptive response has been fed with AP100 from 5dpf to 16dpf. Finally, before imaging, the fish has been fasted for 24h to remove the food residue in the gut. The time points for imaging were labeled by stars. (B) The exact imaging part (in the red box) of the larva.

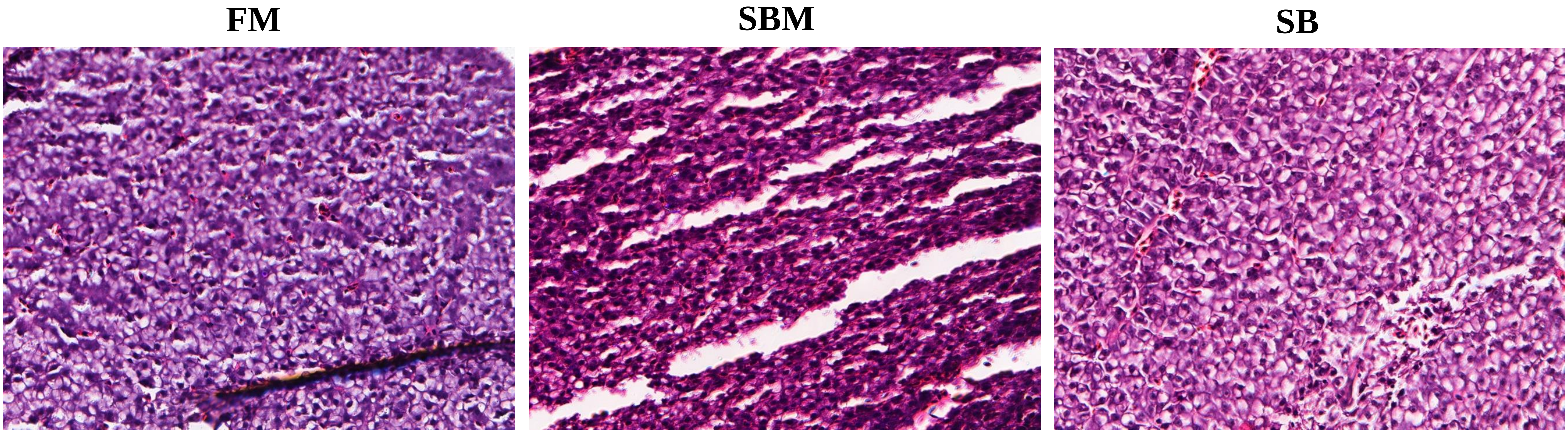

Figure S2 HE staining of liver slice (10um) during the zebrafish SBMIE modeling and seabuckthorn inclusion. FM: fish meal, SBM: soybean meal, SB: seabuckthorn.

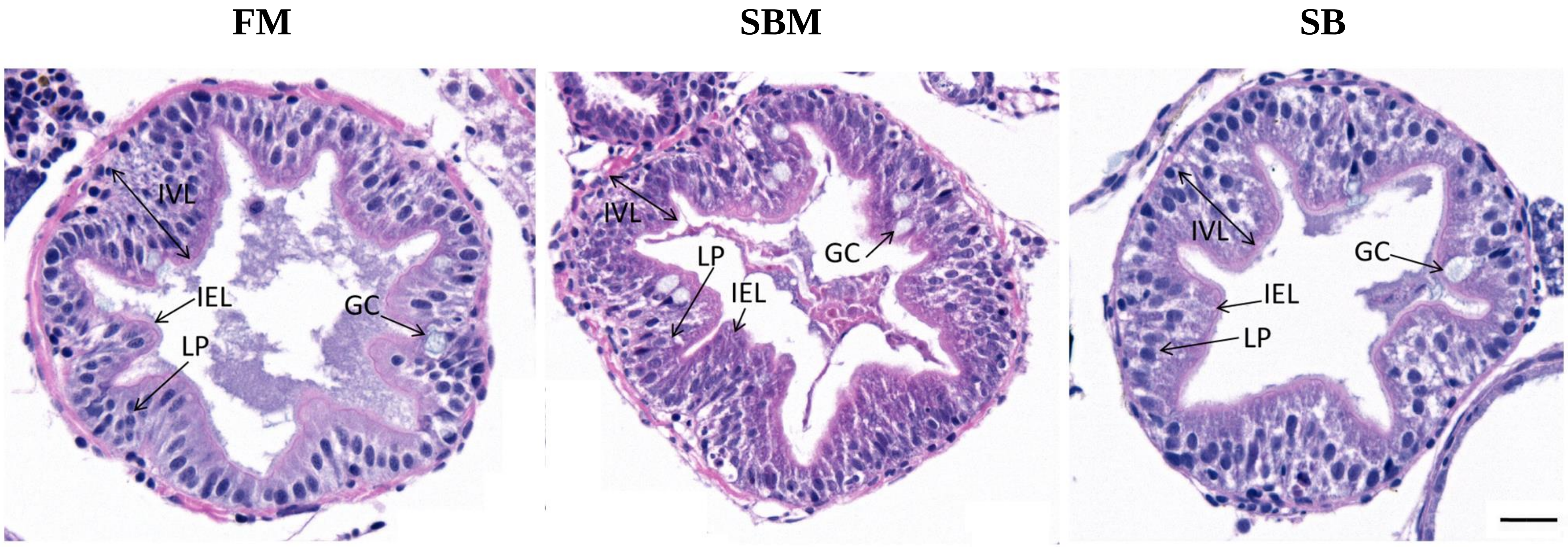

Figure S3 The intestinal villi structure for the last portion of hindgut. Arrows was used to indicate intestinal epithelial layer (IEL), lamina propria (LP) and the goblet cell (GC). FM: fish meal, SBM: soybean meal, SB: seabuckthorn. Bar: 20μm

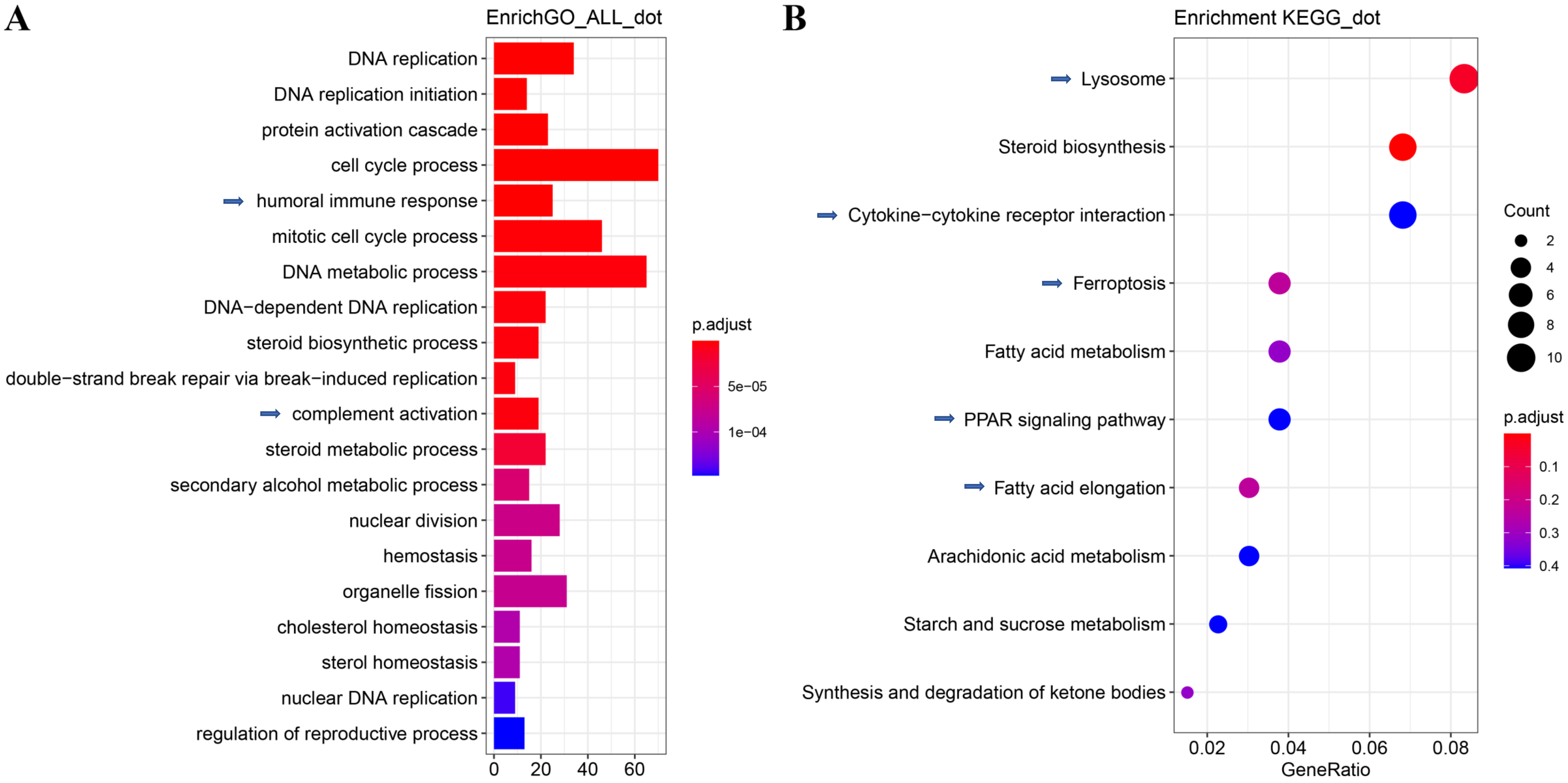

Figure S4 The enrichment analysis of intestinal GO terms (A) and hepatic KEGG pathways (B) using DEGs of the comparison between SBM and FM groups. The immune or inflammation related terms or pathways were labeled with arrows.

(A) FM group

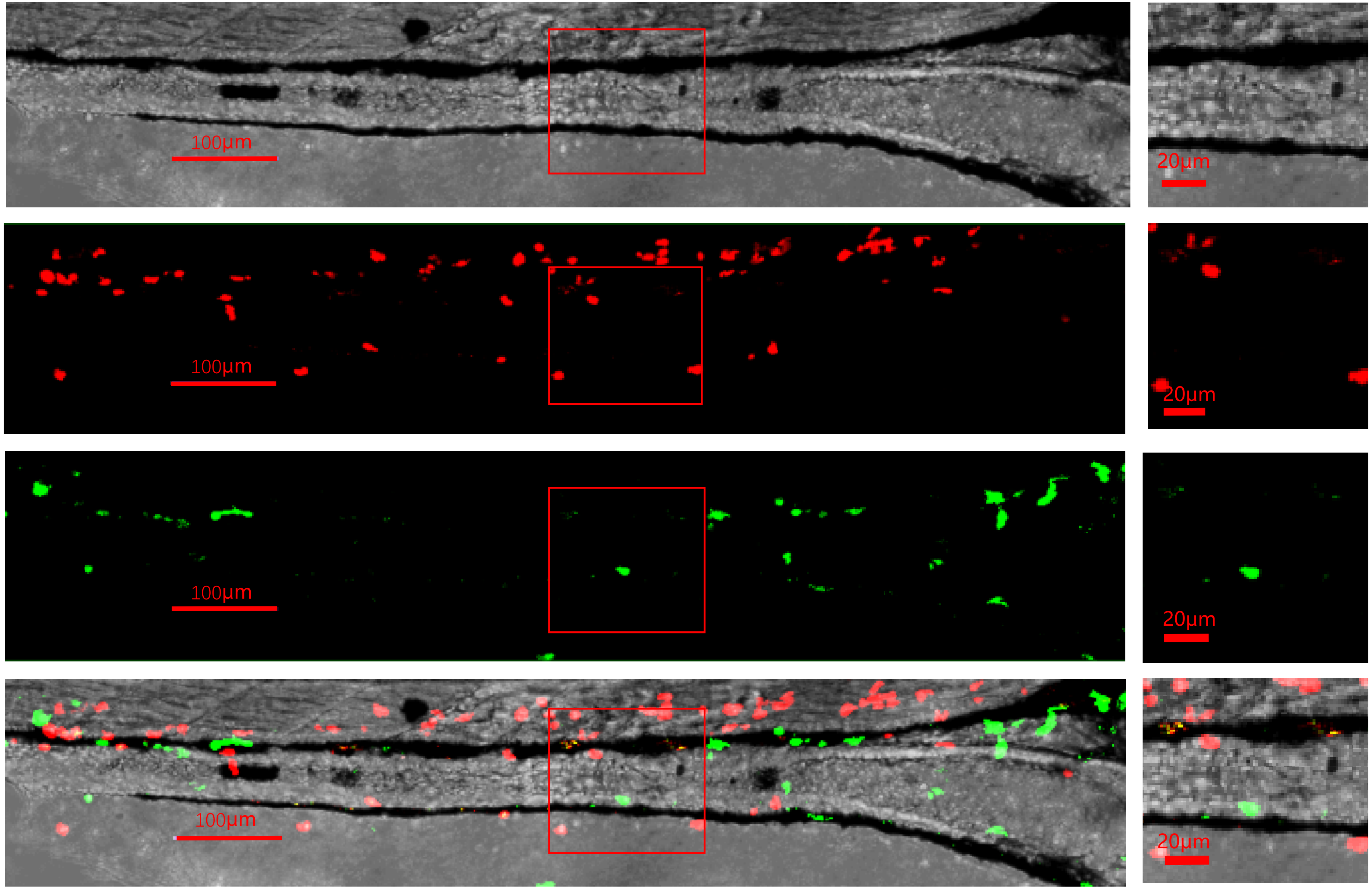

Fig. S5 The original pictures of Figure 6 with high resolution

(B) SBM group

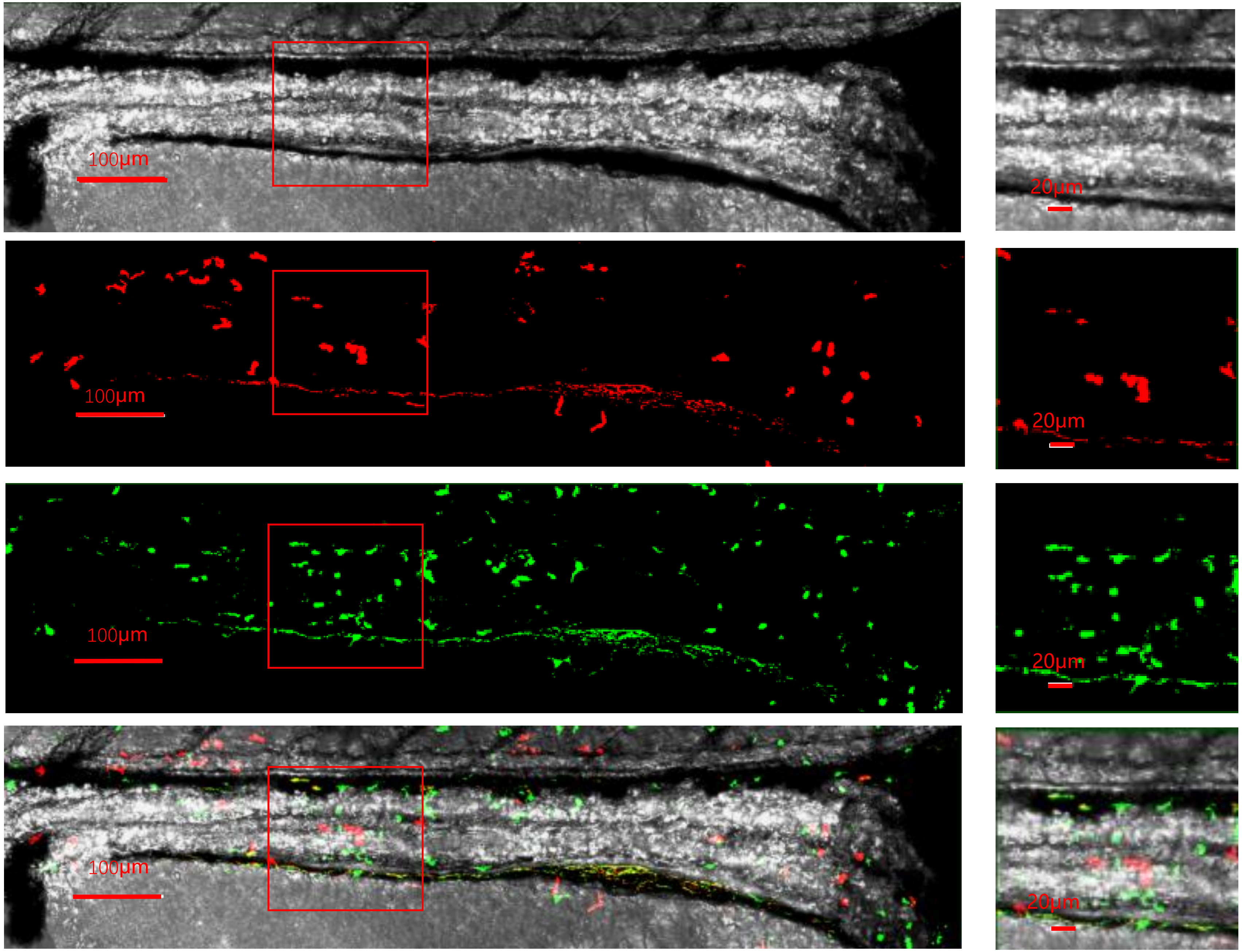

Fig. S5 The original pictures of Figure 6 with high resolution

(C) SB group

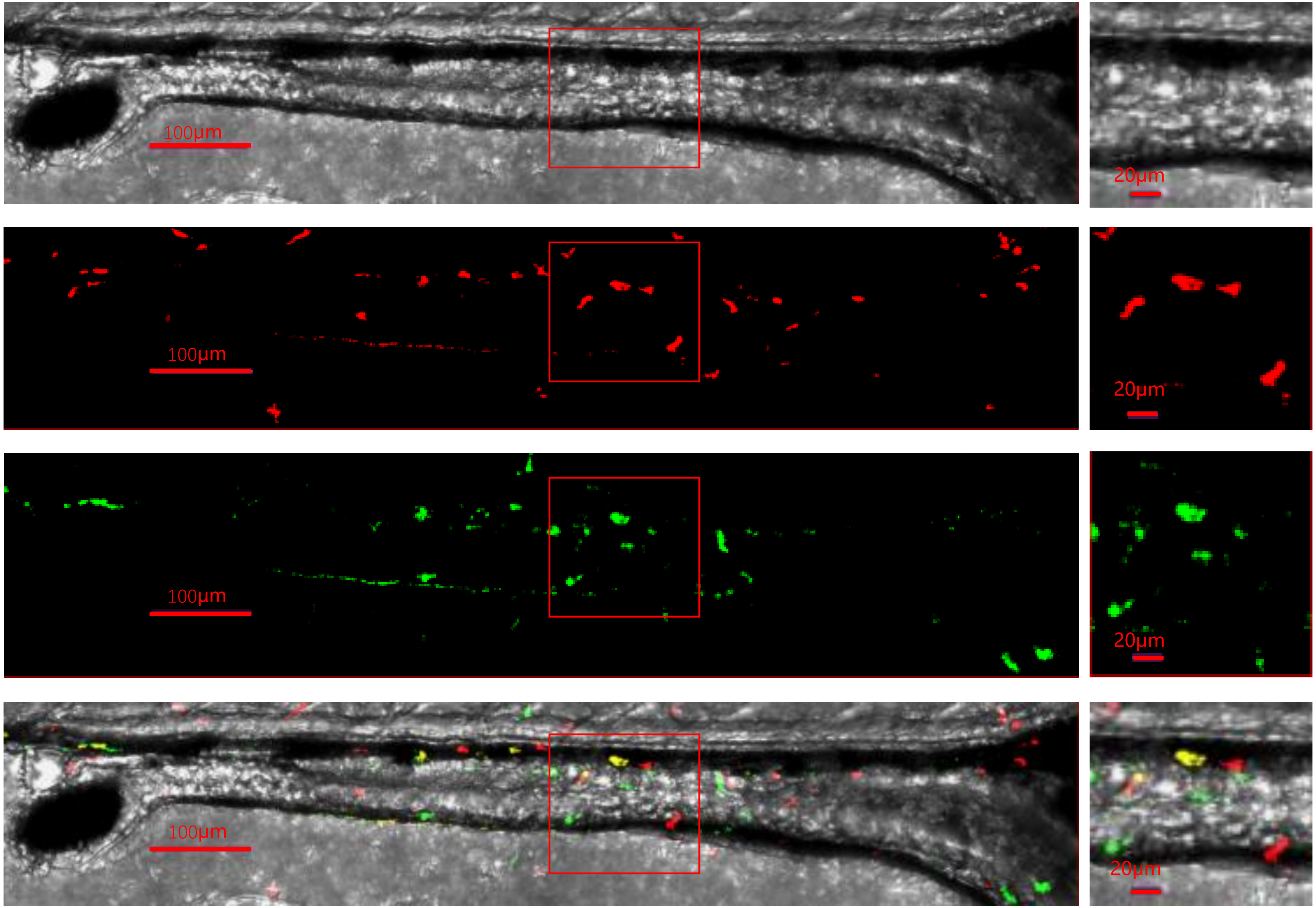

Fig. S5 The original pictures of Figure 6 with high resolution

(A) FM group

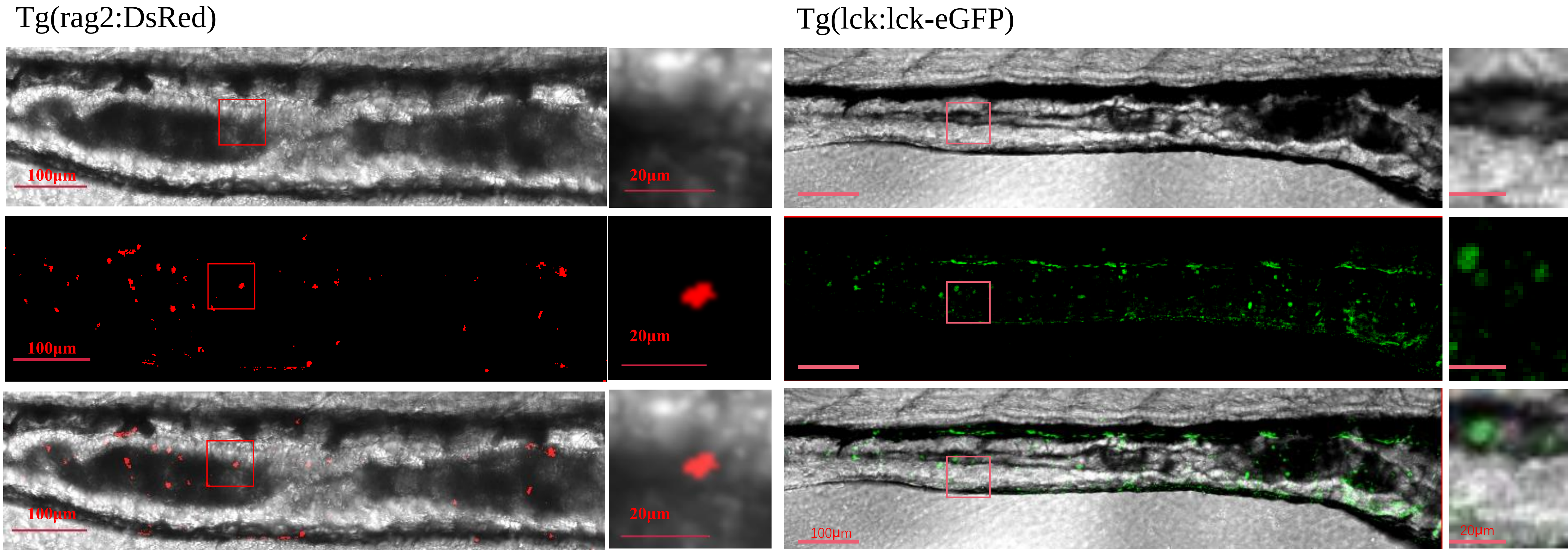

Fig. S6 The original pictures of Figure 7 with high resolution

(B) SBM group

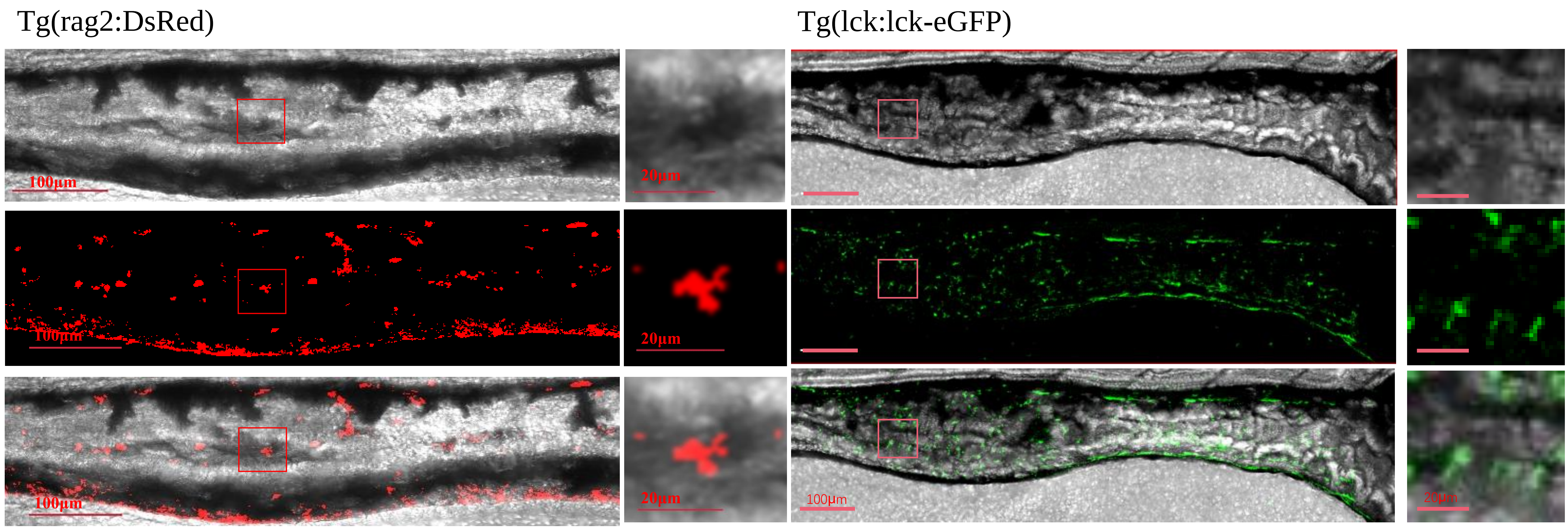

Fig. S6 The original pictures of Figure 7 with high resolution

(C) SB group

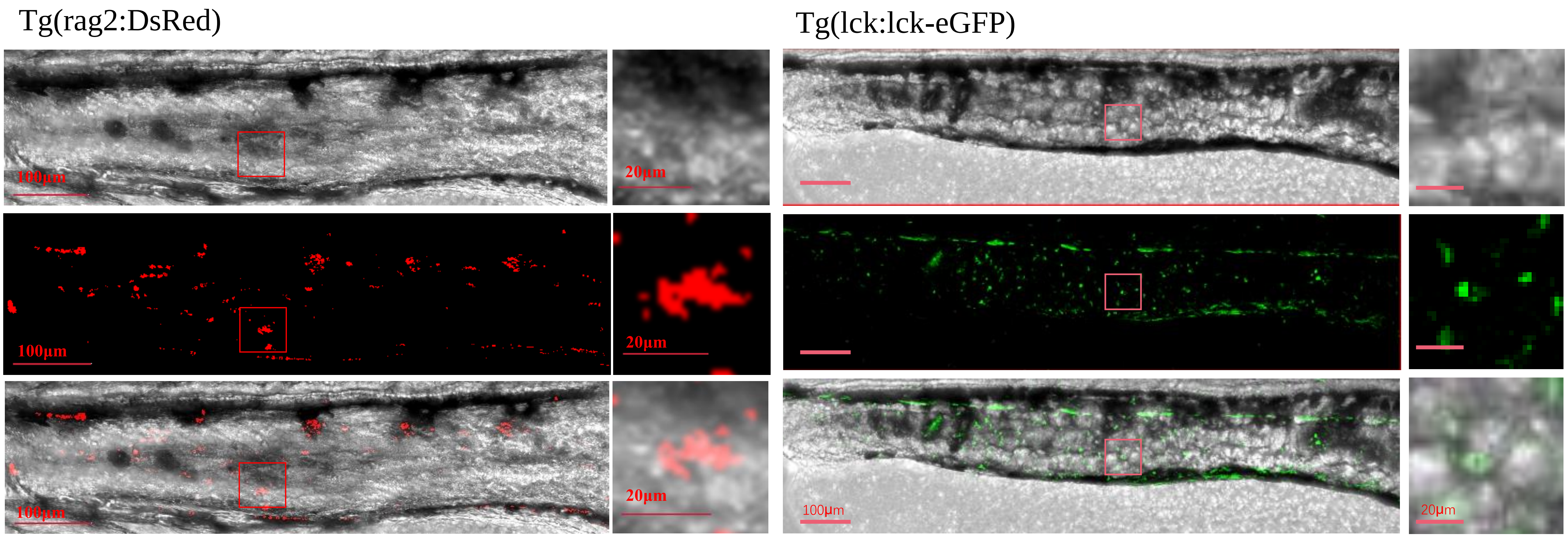

Fig. S6 The original pictures of Figure 7 with high resolution
